# Nubbin isoform antagonism governs *Drosophila* intestinal immune homeostasis

**DOI:** 10.1371/journal.ppat.1006936

**Published:** 2018-03-02

**Authors:** Bo G. Lindberg, Xiongzhuo Tang, Widad Dantoft, Priya Gohel, Shiva Seyedoleslami Esfahani, Jessica M. Lindvall, Ylva Engström

**Affiliations:** 1 Department of Molecular Biosciences, The Wenner-Gren Institute, Stockholm University, Stockholm, Sweden; 2 National Bioinformatics Infrastructure Sweden (NBIS), Science for Life Laboratory, Department of Biochemistry and Biophysics, Stockholm University, Stockholm, Sweden; Massachusetts Institute of Technology, UNITED STATES

## Abstract

Gut immunity is regulated by intricate and dynamic mechanisms to ensure homeostasis despite a constantly changing microbial environment. Several regulatory factors have been described to participate in feedback responses to prevent aberrant immune activity. Little is, however, known about how transcriptional programs are directly tuned to efficiently adapt host gut tissues to the current microbiome. Here we show that the POU/Oct gene *nubbin* (*nub*) encodes two transcription factor isoforms, Nub-PB and Nub-PD, which antagonistically regulate immune gene expression in *Drosophila*. Global transcriptional profiling of adult flies overexpressing Nub-PB in immunocompetent tissues revealed that this form is a strong transcriptional activator of a large set of immune genes. Further genetic analyses showed that Nub-PB is sufficient to drive expression both independently and in conjunction with nuclear factor kappa B (NF-κB), JNK and JAK/STAT pathways. Similar overexpression of Nub-PD did, conversely, repress expression of the same targets. Strikingly, isoform co-overexpression normalized immune gene transcription, suggesting antagonistic activities. RNAi-mediated knockdown of individual *nub* transcripts in enterocytes confirmed antagonistic regulation by the two isoforms and that both are necessary for normal immune gene transcription in the midgut. Furthermore, enterocyte-specific Nub-PB expression levels had a strong impact on gut bacterial load as well as host lifespan. Overexpression of Nub-PB enhanced bacterial clearance of ingested *Erwinia carotovora carotovora 15*. Nevertheless, flies quickly succumbed to the infection, suggesting a deleterious immune response. In line with this, prolonged overexpression promoted a proinflammatory signature in the gut with induction of JNK and JAK/STAT pathways, increased apoptosis and stem cell proliferation. These findings highlight a novel regulatory mechanism of host-microbe interactions mediated by antagonistic transcription factor isoforms.

## Author summary

The numerous human diseases caused by aberrations in intestinal immunity and integrity urge a better understanding of the regulatory interactions that balance the output of host-microbe interactions. In this study, we discovered a novel phenomenon of transcriptional antagonism exerted *via* two isoforms encoded from the same gene. Balanced expression of the two forms was necessary for ensuring normal immune gene expression and maintaining immunological homeostasis in the *Drosophila* gut. We performed genetic manipulations to skew the balance of these isoforms. This resulted in a dysregulated immune system, changed levels of gut bacteria and altered host lifespan. Moreover, when we overexpressed the activating form flies quickly succumbed to oral bacterial infection despite an enhanced immune response. We suggest that antagonistically acting transcription factor isoforms may constitute a general mechanism for adjusting gene expression in various biological processes.

## Introduction

The innate immune system of mammals and insects is regulated by intracellular signaling pathways and transcriptional programs that show remarkable signs of evolutionary conservation. Well-known examples are the Toll/Toll-like receptor (TLR), immune deficiency (IMD)/tumor necrosis factor-α (TNF-α), JAK/STAT and JNK signaling pathways and their respective downstream transcriptional activators, nuclear factor kappa B (NF-κB), STAT and AP-1, which regulate innate immune responses in both *Drosophila* and mammals [1–4]. Pathway activation triggers a vast set of genes that encode effector molecules such as antimicrobial peptides (AMPs) and cytokines, which in mammals also support the induction of adaptive immune responses [5]. The underlying regulation is complex, especially in the intestine and other barrier epithelia that are in constant contact with the commensal microbiota. Improper control of the innate immune system and loss of tissue homeostasis can cause inflammation and other autoimmune diseases, and may lead to system failure and early death [1, 6]. In *Drosophila*, the IMD and Toll pathways and downstream NF-κB homologs, Relish (Rel) [7] and Dorsal-related immunity factor (Dif) [8], are crucial activators of immune genes in response to infection. About 25% of *Drosophila* immune-regulated genes (DIRGs) are, however, expressed independently of these pathways [9]. Additional transcriptional activators [10–13] as well as repressors [14–17] have been implicated in the immune response during specific conditions. However, little is known concerning how such factors compete for the same targets and interact to balance responses and maintain homeostasis.

The POU/Oct transcription factor family is a subclass of the homeodomain proteins present in all metazoans [18] and encompasses crucial regulators of developmental decisions, metabolism, immunity and cancer [19]. Human Oct-1 (POU2F1) was originally discovered as a regulator of adaptive immune responses [20] and has more recently been shown to regulate numerous target genes involved in both innate and adaptive immunity, stress resistance, metabolism and cellular proliferation [21]. In a yeast screen for transcriptional regulators of innate immune response genes, we have previously isolated three candidates from the *Drosophila* POU/Oct family [22]. The Oct-1 homolog, *nub*, was subsequently found to encode a negative regulator (Nub-PD) of NF-κB/Rel-driven immune gene expression in flies [15]. Nub-PD is necessary to control aberrant gene activation as *nub*^*1*^ mutant flies are short-lived and have a severely distorted gut microbiota [23]. Both Oct-1 and Nub regulate target genes by binding to the canonical octamer DNA sequence motif (ATGCAAAT) or variants thereof, via their C-terminal POU_S_ and POU_H_ domains [24–26]. Thus, the DNA-protein interaction surfaces appear conserved, further emphasizing the evolutionary relationship of these ancient transcriptional regulators.

It has earlier been reported that alternative transcript forms of *nub* exist [27]. However, functional studies have, to our knowledge, been focused solely on the Nub-PD isoform. In this study, we demonstrate that *nub* encodes a novel isoform, Nub-PB, which is a strong activator of immune gene expression. Furthermore, both isoforms are expressed in midgut enterocytes and regulate the same immune target genes antagonistically. We show that such tuning of the transcriptional output of Nub target genes is crucial for host immunity, fitness and survival.

## Results

### Nub-PB is an activator of immune gene expression

**Fig 1:**
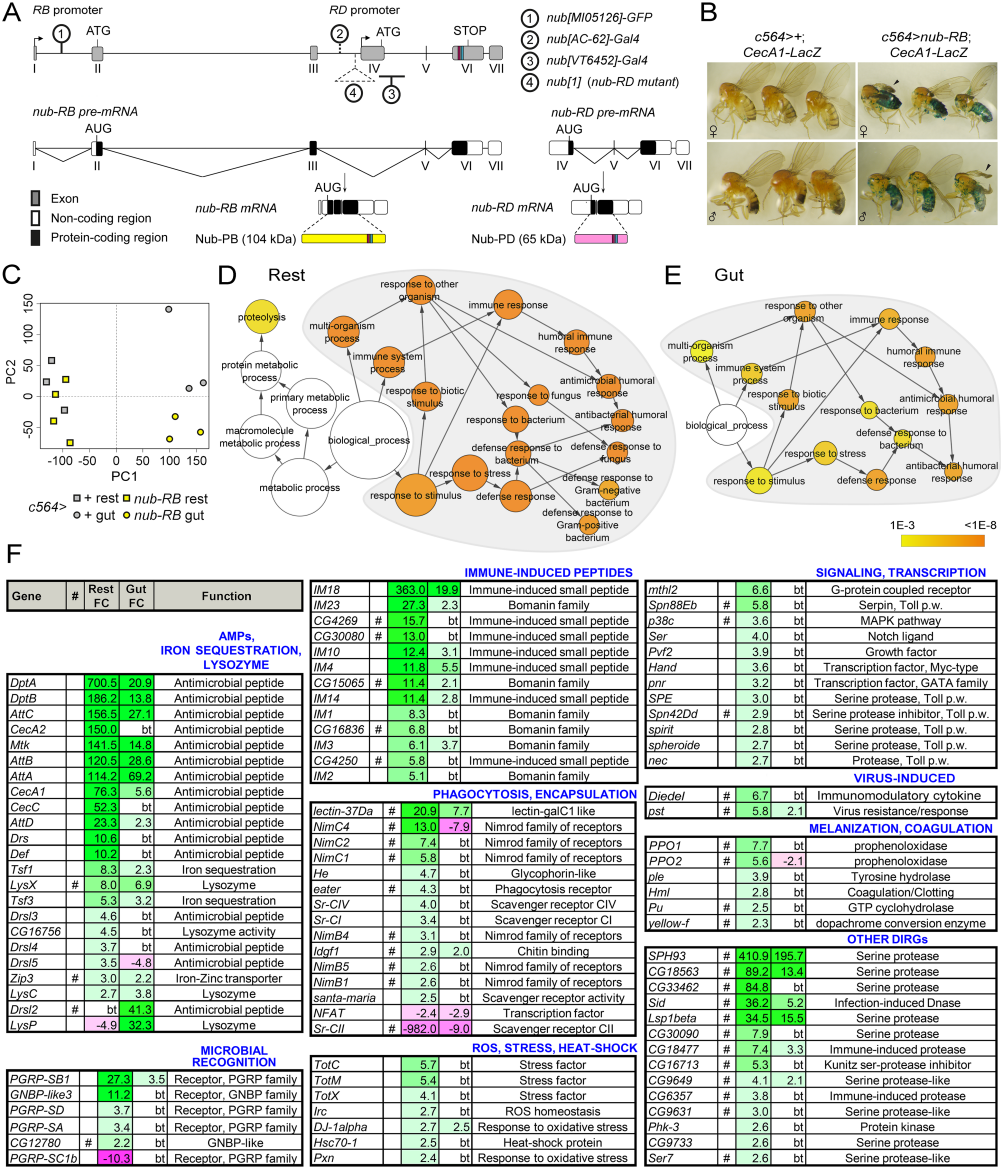
Nub-PB, encoded from the *nub* gene locus, is a strong activator of immune-regulated genes *in vivo*. (A) The *nub* gene locus encodes the *nub-RB* and *nub-RD* transcripts from separate promoters, resulting in Nub-PB (104 kDa; yellow) and Nub-PD (65 kDa; magenta) proteins, respectively. POU_S_ (dark red box), and POU_H_ (turquoise box) are indicated. Nub-specific insertion mutants and Gal4 drivers are indicated with encircled numbers on the gene, and with a key in the right panel. (B) Overexpression of *nub-RB* by c564-Gal4 activates *CecA1-lacZ* expression in fat body (blue). Adult females were maintained at 25 °C prior to fixation and overnight β-gal staining. Arrowheads indicate an unfolded wing phenotype frequently scored in flies with *nub-RB* overexpression. (C-E) Analysis of global mRNA expression profiles in gut tissues and carcasses without heads (rest) from flies overexpressing *nub-RB* (*c564>nub-RB*) in comparison to controls (*c564>+*). (C) Principal component analysis was conducted using all transcripts found to be expressed over background signal in at least one cohort. Samples are colored group-wise according to genotype and subdivided according to tissue, i.e. dissected intestines (gut) and body without head and gut (rest). (D-E) Gene set enrichment analysis (GSEA) of the transcripts found upregulated in “rest” (795 hits; D) and “gut” (159; E). The orange nodes corresponding to different Gene Ontology clusters were found with increasing statistical significance after Benjamini and Hochberg FDR correction (*p*<10^−3^) whereas non-colored nodes were considered non-significant (*p*>10^−3^). Circle sizes reflect the number of genes found within each biological process. The gray-zoned areas highlight statistically enriched nodes relating to immune system processes. (F) Merged list of selected, differentially expressed DIRGs (see S5 Table for full list). Fold changes (FC) reflect mean values from 3–4 independent biological replicates; bt, below signal or significance threshold; # putative DIRGs derived from https://lemaitrelab.epfl.ch/resources; p.w., pathway.

Recent public annotations indicate two promoters of the *nub* gene, which in combination with promoter-specific splicing produce two independent proteins, Nub-PB (104 kDa) and Nub-PD (65 kDa; Fig 1A). Both proteins contain identical C-terminal domains, in which the DNA-binding POU_S_ and homeodomain (POU_H_) are located. Thus, Nub-PB and Nub-PD are expected to bind the same DNA sequences, while abilities for protein-protein interactions are likely to differ due to the distinct N-termini of the proteins. Initial experiments were aimed at detecting and elucidating any immune-regulatory features of Nub-PB. We applied the Gal4-UAS system and constructed a *UAS-nub-RB* line to drive its overexpression in immune competent tissues (midgut and fat body) using the c564-Gal4 driver. To simultaneously detect the ability of Nub-PB to regulate AMP gene expression, we combined *c564>nub-RB* with a *lacZ* reporter construct for *Cecropin A1* (*CecA1*). This resulted in very prominent β-galactosidase activity in the abdominal fat body (Fig 1B). Nub-PB hence appears to function as a transcriptional *activator* of immune effector genes, in stark contrast to Nub-PD.

We carried on by analyzing the global transcriptional profiles of *c564>nub-RB* in comparison to driver controls (*c564>+*, from hereon referred to as wild type [wt]). To enable expression analysis of fat body and gut independently, mRNA from the fly body without head and gut (“rest”) and the digestive system (“gut”) were extracted and analyzed separately. The raw data were normalized, preprocessed and filtered to remove genes that were not expressed above the detection level (S1 Table; Methods). Unbiased principal component analysis (PCA), correlation analyses and hierarchical clustering showed that the tissue variable (“rest” versus “gut”) accounted for the largest distinction, as expected (PC1, explains 47% of the variance; Figs 1C and S1A). The PCA furthermore indicated that genotype accounted for the second largest separation in the dataset (PC2 explains 9.5%; most apparent in “gut”). After adjusting *p*-values for a false discovery rate of 1%, 1177 (rest) and 545 (gut) probes indicated significant differential expression (S1 Table), of which 132 were in common for both tissues (S1B Fig, S2 Table). Gene set enrichment analyses (GSEA) according to “Biological process” of upregulated transcripts in respective tissue resulted in a single major GO cluster encompassing various aspects of immunity (Fig 1D and 1E, S3 Table). Of transcripts that were found downregulated, the only significant subnode in “rest” was “proteolysis” (also significant for upregulated probes in “rest”), whereas those from gut samples formed GSEA clusters of several overlapping cellular and developmental processes such as cell fate commitment, organ development, Notch signaling pathway and sensory responses along with GO categories of antibacterial defense and transcriptional regulation, suggesting a multifaceted role of Nub-PB in this organ (S2 Fig, S3 and S4 Tables). To detect additional DIRGs, the dataset was manually curated and run against a collection of putative immune genes (https://lemaitrelab.epfl.ch/resources, accessed February 2015), which yielded 152 DIRGs in total (Fig 1F, S5 Table). Overall, a striking coherence with well-characterized DIRGs typical for activated Toll, IMD and JAK/STAT pathways was observed. In summary, the global expression analysis demonstrated a broad immune activation following *nub-RB* overexpression, which further indicates that Nub-PB is a transcriptional activator of immune genes.

### Nubbin isoforms regulate the same genes in an antagonistic manner

**Fig 2:**
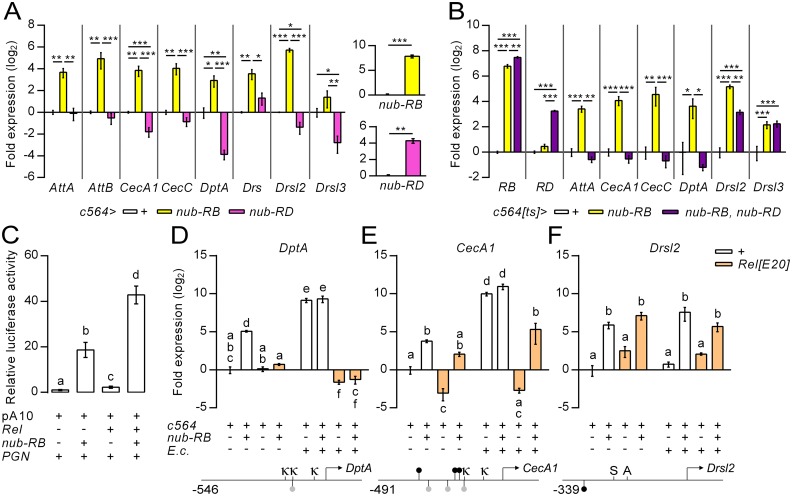
Nub-PB and Nub-PD regulate the same genes in an antagonistic and Relish-dependent manner. (A) Quantification of relative mRNA expression from selected AMP genes along with each isoform of *nub* (inserts), from whole fly extracts using RT-qPCR following overexpression of *nub-RB* (yellow bars) or *nub-RD* (magenta bars) using *c564>Gal4* (means ± SE, *N* = 3). (B) Quantification of relative mRNA expression from selected AMP genes along with each isoform of *nub* (inserts), from whole fly extracts using RT-qPCR following overexpression of *nub-RB* (yellow bars) or both (purple bars) using *c564>Gal4*^*ts*^ (means ± SE, *N* = 4). (C) Transfection of mbn-2 cells with a pA10-*CecA1-luc* construct (15) with or without expression plasmids for *nub-RB* and/or *Rel*. Cells were subsequently stimulated with bacterial peptidoglycan (in the form of crude LPS) to induce a robust immune response. The graph depicts mean values of relative luciferase activity ± SE (*N* = 3). (D-F) Quantification of relative mRNA expression from selected AMP genes using RT-qPCR following c564-driven overexpression of *nub-RB*, with or without a Rel mutant background (*Rel*^*E20*^, orange bars) compared to controls and with or without systemic infection with *E*. *cloacae* (means ± SE; *N* = 3). Lower panels display proximal promoter regions of *DptA*, *CecA1* and *Drsl2*. Oct (ATSBAAAW; ●), and Oct-like (ATTCAAAT; ●) motifs are depicted in the proximal promoter region of each gene [15]. κ = κB-site; S = Stat92E-site; A = AP-1 site. Bars represent means ± SE. Asterisks and distinct letters denote significant differences (*p<0.*05*; **p<0.*01*; ***p<0.*001*; distinct lettering, *p*<0.05).

A comparative analysis of the differentially expressed genes in response to Nub-PB overexpression (this study) and *nub*^*1*^ mutant flies (disrupts Nub-PD, but not Nub-PB expression) [15, 27] revealed an extensive overlap as 65 immune-regulated genes were upregulated in both transcriptional profiles (S6 Table). Out of the most highly expressed immune-process genes in “rest” (FC>10, 26 genes), 25 were upregulated at least 2-fold in *nub*^*1*^ mutants. To compare the capacity of Nub-PB and Nub-PD to regulate AMP genes, c564-driven overexpression of either transcriptional regulator was performed in parallel experiments (Fig 2A). In validation of the transcriptional profiling, overexpression of *nub-RB* promoted strong upregulation of all eight assayed AMP genes (*AttA*, *AttB*, *CecA1*, *CecC*, *DptA*, *Drs*, *Drsl2* and *Drsl3*) from whole fly extracts. Conversely, overexpression of *nub-RD* decreased *CecA1*, *DptA*, *Drsl2* and *Drsl3* significantly compared to controls. We next evaluated the combined effect of the two isoforms in co-overexpression assays. To circumvent developmental and secondary effects, a temperature sensitive (ts) *c564-Gal4; Tub-Gal80* driver line was applied. This resulted in expression levels of *AttA*, *CecA1*, *CecC* and *DptA* similar to those in the control cohort (Fig 2B), indicating that the two Nub isoforms overall neutralize each other’s activity. Co-expression did not dampen the increased expression of *Drsl3*, whereas *Drsl2* was partially restored to control levels, suggesting that Nub-PB induces these targets with greater affinity than the corresponding repression mediated by Nub-PD. We conclude that the Nub isoforms are able to regulate the expression of several AMP genes in an antagonistic manner.

### Nub-PB and Relish synergistically activate immune genes

It has previously been shown that Nub-PD is a repressor of Rel-target genes in the absence of infection [15]. The observation that Nub-PB overexpression activates the same gene set (S6 Table) prompted us to explore its putative cooperation with Rel. Co-transfection of mbn-2 cells with *nub-RB* and *Rel* induced *CecA1-luciferase* expression significantly stronger (42.8-fold) than single transfections (*nub-RB*, 18.6-fold; *Rel*, 2.2-fold), which also indicated a synergistic effect (Fig 2C). We therefore hypothesized that Nub-PB acts as a co-activator of Rel. To investigate this, AMP expression was assayed in whole flies following overexpression of *nub-RB* in a *Rel* mutant (*Rel*^*E20*^) or wild type background (Fig 2D–2F). To induce a robust IMD pathway response, separate cohorts were subjected to systemic infection with *Enterobacter cloacae* β12. Three AMP genes were assayed based on their known dependency of Rel and proximal Nub/Oct-sites: *DptA* (Rel-dependent, one Nub/Oct-site), *CecA1* (Rel-dependent, several Nub/Oct-sites) [15, 28] and *Drsl2* (Rel-independent, one Nub/Oct-site) [29]. Of note, binding of Nub to the proximal promoter region of *DptA* and *CecA1* has been demonstrated biochemically [15]. Overexpression of Nub-PB was sufficient to drive expression of *CecA1*, but not *DptA*, in absence of Rel and independent of infection status (Fig 2D and 2E). *Drsl2*, specifically expressed in the gut *via* the JAK/STAT pathway [29] was, as expected, not affected by the *Rel*^*E20*^ mutant background (Fig 2F). Taken together, the data suggest that Nub-PB can influence AMP gene transcription both independently of, and together with Rel.

### An Oct sequence cluster is required for full *Cecropin A1* induction by Nub-PB *in vivo*

**Fig 3:**
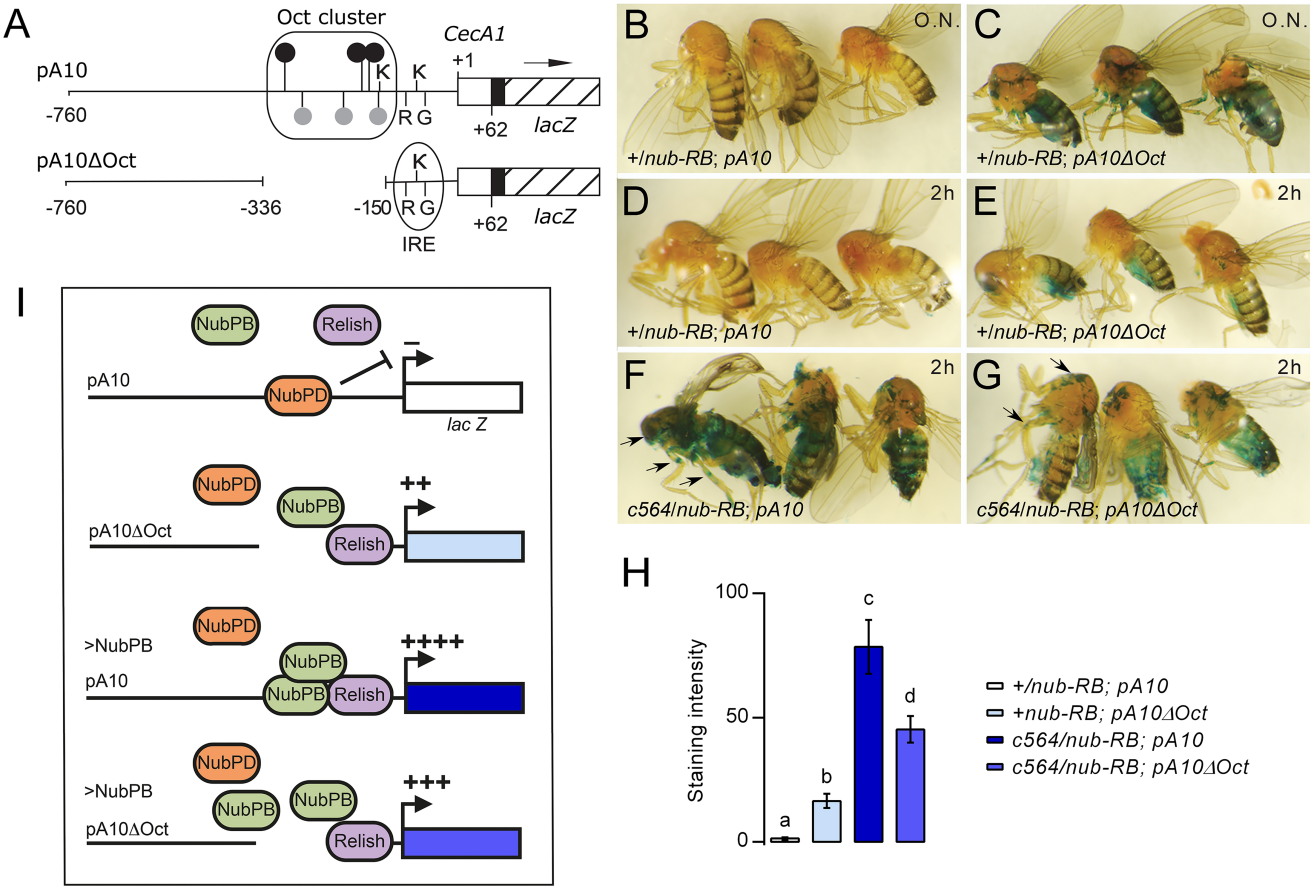
The Oct motif cluster is required for both Nub-PD-dependent repression and Nub-PB-driven activation of the *CecA1-lacZ* reporter. (A) Schematic representation of the *CecA1-lacZ*-constructs carried by transgenic fly strains. The pA10 construct contains 760 bp of 5’ upstream region from the *CecA1* gene (horizontal line), and 62 bp of 5’ UTR (open box) fused to an SV40 leader (filled box), providing a translational start site in frame with the *E*. *coli lacZ* coding sequence (hatched box) [15, 47]. Numbers refer to positions relative to the transcription start site (+1). Location of regulatory sequence motifs, as indicated by symbols and letters, is in scale. A previously characterized infection-induced response element (IRE) contains a κB-like site (“κ”), GATA site (“G”) and R1 site (“R”), and an additional κB site located 5′ in close proximity of the IRE [56]. The cluster of Oct sequence motifs contains several consensus Oct sequences (●) and Oct-like (●) sequences [15]. The *pA10*Δ*Oct* construct has an internal deletion of the whole Oct cluster (−336 to −150) but is otherwise identical to the *pA10* construct. (B-G) *CecA1*-driven β-gal staining in fat body and other tissues in female flies carrying either the *pA10* construct (B, D, F) or the *pA10ΔOct* construct (C, E, G), in combination with *c564-Gal4* driven overexpression of *nub-RB* (F, G) or in control flies without *c564-Gal4* (B-E), with the genotypes as indicated. Incubation with the β-gal substrate X-gal was either carried out overnight (O.N.) or for 2 h (D-G). Note the reporter gene expression in the thorax region and legs (F-G) as indicated by arrows. (H) Estimate of X-gal intensity, by conversion of blue pixels in images to 8-bit grayscale values ranging from 0 (white, no staining) to 255 (black) (*N* = 6). Group letters denote significantly different cohorts (*p*<0.05). (I) Schematic model of *CecA1*-*lacZ* regulation by Nub-PD and Nub-PB via the Oct sequence cluster. Nub-PD (orange) binds to the Oct cluster and prevents *CecA1* expression in healthy flies, also in the presence of Relish (blue) and Nub-PB (green) (B and D). In constructs lacking the Oct cluster (*pA10ΔOct*), Nub-PD cannot bind and repress the *CecA1* promoter, which leads to moderate activation (++) of reporter gene expression (C and E). Overexpressed Nub-PB will compete with Nub-PD for binding to the Oct cluster and hyperactivates (++++) expression from the intact promoter (F), while the activation is not as prominent (+++) in constructs lacking the Oct cluster (G).

The proximal promoter region of *CecA1* contains a cluster of six Oct/Oct-like sites required for Nub-PD mediated repression of a *CecA1-luciferase* reporter construct *in vitro* [15]. To investigate the requirement of this cluster for the regulatory capacity of Nub-PB and Nub-PD *in vivo*, the expression of different reporter constructs was analyzed in female (Fig 3) and male flies (S3 Fig). As expected, flies carrying the *CecA1-lacZ* construct with an Oct cluster deletion (pA10ΔOct; Fig 3A) displayed a derepressed and hence stronger reporter gene expression in fat body than flies with the complete upstream region (Figs 3B, 3C, 3H and S3A and S3B). Since deletion of the cluster promoted reporter gene expression *per se*, the incubation time was decreased to circumvent saturation of the response (Figs 3D–3G and S3C–S3F) and combined with c564-driven overexpression of Nub-PB (Figs 3F, 3G and S3E and S3F). The full-length pA10 *CecA1-lacZ* reporter gene responded strongly to Nub-PB overexpression (Fig 3F and 3H; also shown in Fig 1B). Overexpression combined with the Oct cluster deletion did, however, not promote full transcriptional activation (Fig 3G and 3H). Still, an intermediate level of β-gal reporter gene expression was observed, significantly stronger than in the Oct deletion control (Fig 3E and 3H), suggesting that the *CecA1* locus may contain additional Nub target sequences outside the pA10ΔOct region. We conclude that both Nub isoforms require the Oct cluster for accurate regulation of the *CecA1* gene (Fig 3I).

### Spatial co-localization of Nub isoforms

**Fig 4:**
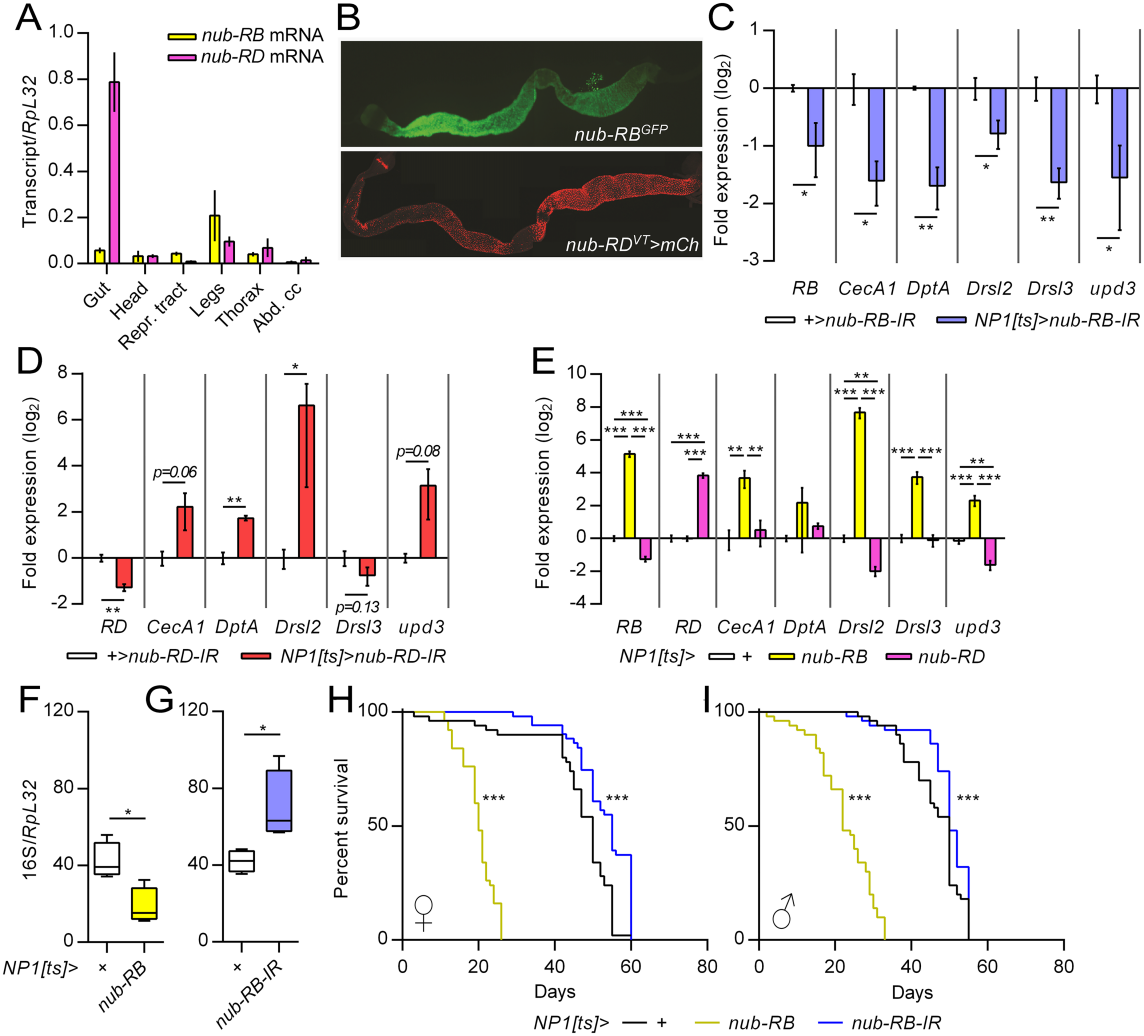
Nub isoforms regulate immune genes in midgut enterocytes. (A) Relative transcript levels of *nub-RB* (yellow) and *nub-RD* (magenta) in extracts of adult body parts and tissues as indicated were quantified by RT-qPCR (*N* = 3). (B) Expression of reporter genes in adult female guts under the control of the RB-promoter (*nub*^*MI05126*^*-GFP*; upper panel) and PD-promoter (*nub*^*VT6452*^*-Gal4>mCherry*; lower panel). (C-D) RT-qPCR of selected immune genes from midgut extracts following RNAi-mediated knockdown of *nub-RB* (C; blue) or *nub-RD* (D; red) in midgut enterocytes using *NP1-Gal4*^*ts*^ and compared to controls (white); *N* = 3. (E) RT-qPCR of AMP genes and *upd3* from midgut extracts following *NP1*^*ts*^-driven overexpression of either *nub* isoform, compared to controls. (F-G) qPCR analysis of bacterial 16S rDNA relative to host genomic *vvl* levels (internal control) from dissected midguts following overexpression (F; yellow) or RNAi of *nub-RB* (G; blue) in midgut enterocytes by *NP1-Gal4*^*ts*^, relative to controls (white). (H-I) Lifespan assays of female (H) and male (I) flies after overexpression (yellow) or downregulation (blue) of *nub-RB* (for statistics, see S6 Fig). Bars reflect means ± SE and asterisks denote significant differences (*p<0.*05*; **p<0.*01*; ***p<0.*001*).

Transcription factor antagonism requires a co-localized expression. We explored the spatial distribution *in vivo* of *nub-RB* and *RD* transcripts from dissected tissues of adult male flies (Fig 4A). Notably, the RNA levels of *nub-RD* exceeded that of *nub-RB* in the gut, whereas expression of the two appeared roughly similar in other tissues. To further investigate the spatial expression, we applied transgenic reporter lines specific for either the *nub-RB* or *nub-RD* transcript. A dual isoform marker line (*nub-RB*^*GFP*^, *nub-RD*^*AC-62*^*>mCherry*) was applied to visualize the spatial expression of both isoforms in parallel (S4 Fig). Prominent dual fluorescence was observed in larval wing discs, leg discs and foregut, with varying degree of overlap, whereas only *nub-RD>mCherry* was expressed in the larval brain (S4A–S4C Fig). For adult expression, tissues from *nub-RB*^*GFP*^ and *nub-RD*^*AC-62*^*>mCherry* flies were studied separately. Fluorescence was observed in wing veins and leg joints with either line, whereas mainly *nub-RD* appeared expressed in the abdominal fat body or adjacent tissues (S4D–S4I Fig). A strong GFP signal was observed throughout the midgut of *nub-RB*^*GFP*^ flies (Fig 4B) while *nub-RD*^*AC-62*^-driven mCherry expression was present only in the anterior region and faded within days in newly eclosed adult flies (S4A’ Fig). Therefore, additional *nub-RD-Gal4* driver lines were screened and especially *nub-RD*^*VT6452*^ drove prominent mCherry expression in the midgut (Figs 1A and 4B). For the purpose of the present work, we conclude that the isoforms are expressed to varying degrees in immune competent tissues and thereby should be able to act as transcriptional antagonists in a competitive manner.

### Nub isoforms are expressed in enterocytes and regulate midgut AMP expression, bacterial load and adult life span

In contrast to the observed isoform transcript levels in Fig 4A, Western blot experiments on midgut and carcass extracts using a non-discriminatory Nub antibody yielded an overall stronger band for Nub-PB (S5A Fig) [15]. This suggests that additional regulation occurs post-transcriptionally. Since *nub-RD* mutant flies exhibit chronic immune activation and microbial dysplasia in the gut [15, 23], we hypothesized that this isoform is required to suppress aberrant activity of Nub-PB in these tissues. In fact, Nub immunostaining is commonly used to mark the nucleus of gut enterocytes [30], which suggests that at least one of the isoforms is strongly expressed in this cell type. The enterocyte-specific driver NP1-Gal4^ts^ was therefore applied to drive RNA interference (RNAi) of *nub-RB* (*nub-RB-IR*) in adult flies. This resulted in a roughly two-fold decrease of the targeted transcript accompanied by a significant reduction of *Drsl2*, *Drsl3*, *DptA*, *CecA1* and *upd3* in midgut tissues (Fig 4C). The latter encodes an infection-inducible cytokine and ligand of the JAK/STAT pathway [31, 32] and was found upregulated 4.8-fold in the gut, albeit below the signal threshold in the global transcriptional profile (S1 Table). The RNAi was further confirmed at the protein level, as Nub-PB decreased 2-fold in extracts from whole flies, strongly indicating that enterocytes constitute a major source of this isoform (S5B Fig). Similar effects were observed with *c564-Gal4*^*ts*^ in the midgut, but not in the abdominal carcass (encompassing the fat body), suggesting that the endogenous Nub-PB acts as a positive regulator of AMPs specifically in the midgut (S5C and S5D Fig). Conversely, RNAi-mediated downregulation of *nub-RD*, using a similar assay as in Fig 4C, resulted in an overall increased expression of the same set of genes (Fig 4D). Comparative overexpression of the two isoforms in gut enterocytes confirmed their opposite regulatory effects on immune genes, previously observed in whole flies (Fig 2A and 2B), as *Drsl2* and *upd3* were strongly up- and downregulated in midgut extracts following *nub-RB* and *nub-RD* overexpression, respectively (Fig 4E). *CecA1* and *Drsl3* levels were increased by *nub-RB* but unaltered by *nub-RD* whereas neither overexpression affected *DptA* significantly. Interestingly, *NP1*^*ts*^>*nub-RD* decreased the expression of *nub-RB* 2.4-fold, but not *vice versa*. To investigate whether Nub-PB expression affected the gut bacterial load, we performed a qPCR analysis of the relative 16S rDNA levels from midgut extracts. *NP1*^*ts*^-driven overexpression of *nub-RB* resulted in a ~56% reduction of bacteria after 24 h (Fig 4F). Conversely, RNAi using the same driver increased bacterial loads by 67% (Fig 4G). The same set of flies displayed striking lifespan phenotypes as *nub-RB* overexpression significantly shortened median longevity (females, 19.3; males, 20.3 days; average from three individual experiments) relative to controls (females, 46.2; males 45.8 days), whereas its downregulation increased longevity (females, 52.3; males, 48 days) (Figs 4H, 4I and S6A–S6C). Overexpression of *nub-RD* resulted in longer lifespan (females, 29; males 33 days) compared to that of *nub-RB*, but shorter than controls (S6B and S6C Fig). Surprisingly, antibiotic supplementation of the diet enhanced longevity of *nub-RB*-overexpressing flies (females 15.8%, males 28.9%) but not those of *nub-RB* RNAi (females -3.1%; no change in males), suggesting that the microbial composition, rather than loads influences host lifespan (Figs 4F, 4G and S6B and S6D). The germ-free conditions enhanced female (6.8%), but not male controls. Conversely, germ free *nub-RD* overexpressing males displayed enhanced longevity at early time points, but were overall not significantly benefited, whereas females were equally long-lived in comparison to conditionally reared flies. Together, these findings demonstrate that Nub isoforms regulate the expression of midgut AMPs in opposite manners and that Nub-PB expression correlates with gut microbiota and host lifespan.

**Fig 5:**
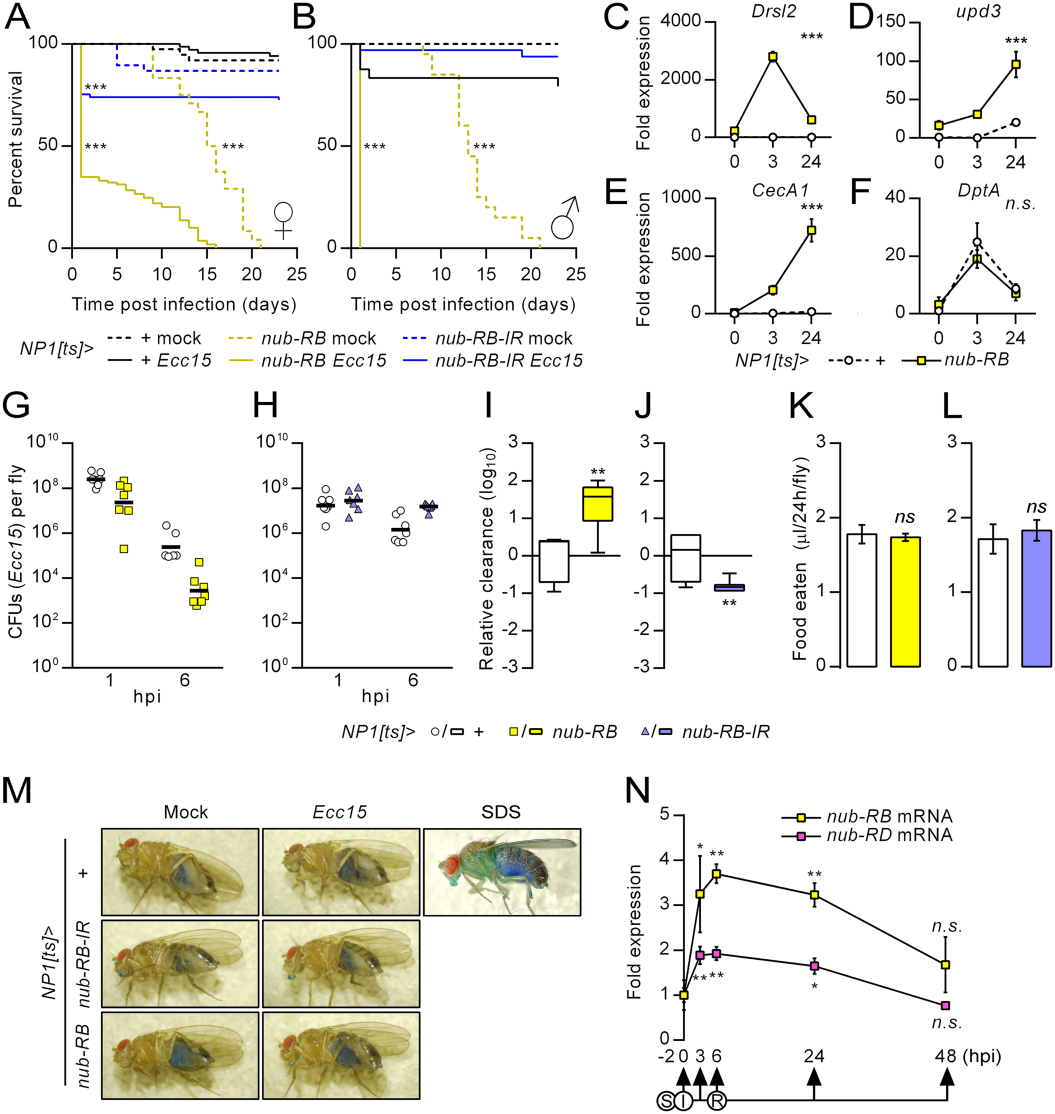
Enterocyte-specific *nub-RB* overexpression renders flies hypersensitive to infection despite increased AMP levels and enhanced pathogen clearance. (A-B) Survival curves of mock-infected (dashed lines) or *Ecc15* orally infected female and male flies (filled lines), after simultaneous overexpression (yellow) or downregulation of *nub-RB* (blue) compared to controls (black) in midgut enterocytes using *NP1-Gal4*^*ts*^. Females mock, *n* = 24–38; females infected, *n* = 67–109; males mock, *n* = 20; males infected, *n* = 24–32. The graphs are representative of two independent experiments. (C-F) Quantification of relative mRNA expression from whole fly extracts, in uninfected flies or following 3 or 24 h post oral *Ecc15* infection, with (yellow rectangles) or without (white circles) simultaneous overexpression of *nub-RB* in midgut enterocytes. Relative levels were normalized to those of uninfected control flies (set to 1). (G-H) Colony forming units from control flies (white circles), flies with overexpression (yellow squares), or downregulation of *nub-RB* (blue triangles), orally infected with *Ecc15*-*GFP*. Serial dilutions were prepared from whole fly extracts and plated at 1 and 6 hpi. After 1 hpi, remaining flies were transferred to regular food without bacteria. (I-J) Box-Whisker plots (min-max, including outliers) of bacterial clearance from 1 to 6 hpi relative to control flies. (K-L) Capillary feeding assay of the denoted genotypes (*N* = 5). (M) Representative images of flies from the Smurf assay, initiated at 6 hpi with *Ecc15* or control food (Mock) followed by flipping flies onto regular fly food supplemented with blue food dye. No smurfs were recorded within the assayed period (up to two weeks post infection). As control, 5% SDS-supplementation of the food resulted in a high penetrance of smurfs within 3 days (note the blue head and thorax). (N) Post-infection time series of the relative midgut expression of *nub* transcript forms relative to those at 0 hpi, determined by RT-qPCR (*N* = 3). Flies (*w*^*1118*^) were starved (S) for 2 h prior to infection (I) on an *Ecc15*-diet until 6 hpi and subsequently transferred to regular food vials for recovery (R). Bars and boxes denote means ± SE and asterisks (*p<0.*05*; **p<0.*01*; ***p<0.*001*).

### Dysregulation of *nub-RB* affects survival to oral infection

To investigate the role of Nub-PB after bacterial challenge, we performed oral infections using *Erwinia carotovora carotovora 15* (*Ecc15*), a well-characterized bacterium with generally low oral pathogenicity in adult flies. Strikingly, overexpression of *nub-RB* caused a hypersensitive phenotype as all males and roughly 70% of the females succumbed within a day post infection (Fig 5A and 5B). Interestingly, the median lifespan of the remaining overexpressing female survivors after bacterial exposure was 10 days, suggesting that death occurs primarily due to acute effects. RNAi directed against *nub-RB* caused a significant female (~25%), but not male, mortality during the acute stage of infection compared to infected driver control flies. We next explored the induction of the immune response mediated by the combined overexpression of *nub-RB* and *Ecc15* infection (Fig 5C–5F). At three hours post infection (hpi), *Drsl2* expression was upregulated by three orders of magnitude relative to the similarly infected driver control cohort, while at 24 hpi, levels were more comparable to those of the uninfected overexpression cohort (Fig 5C). This indicates a rapid and transient hyperinduction of this gene by the combined effect of overexpression and infection. The induction of *upd3* and *CecA1* was also strongly enhanced by the combined effect of overexpression and infection, with peak expressions occurring later than for *Drsl2* (Fig 5D and 5E). Conversely, and in line with Fig 4E, *DptA* was not affected by *nub-RB* overexpression with NP1 (Fig 5F). We hypothesized that such a strong effect on the immune response would likely be detrimental for the host, while at the same time enhance clearance of the infection. In line with this, enterocyte expression levels of *nub-RB* correlated with the relative clearance of *Ecc15* compared to controls (Fig 5G–5J). In comparison, overexpression of *nub-RD* caused a moderate, non-significant decrease in clearance (S7A and S7B Fig). To exclude a confounding effect of feeding rate, a capillary feeding assay was performed where the different genotypes were found to consume similar amounts to controls (Figs 5K, 5L and S7C). Proinflammatory responses in the gut could potentially cause epithelial damage and ultimately cause gut leakage. To test this, we applied the Smurf assay [54] but did not observe any flies turning blue, neither from genotype, infection, nor the combination of both, suggesting that death occurs due to other causes (Fig 5M). We also observed upregulated levels of both isoforms in the midgut of orally infected flies, albeit stronger for *nub-RB* than *nub-RD* (Fig 5N). This suggests that the balance is temporarily skewed towards the activating function of Nub-PB during the acute stage of infection. Following recovery on regular fly food, the expression of both isoforms returned to pre-infection levels around 48 hpi, indicating a pattern typical for transiently induced DIRGs. Taken together, these data indicate that Nub-PB is involved in the midgut immune response to ingested *Ecc15* and that the activity of this transcription factor requires tight control to avoid detrimental effects on the host.

### Enterocyte-specific overexpression of *nub-RB* promotes a proinflammatory signature and epithelial renewal in the midgut

**Fig 6:**
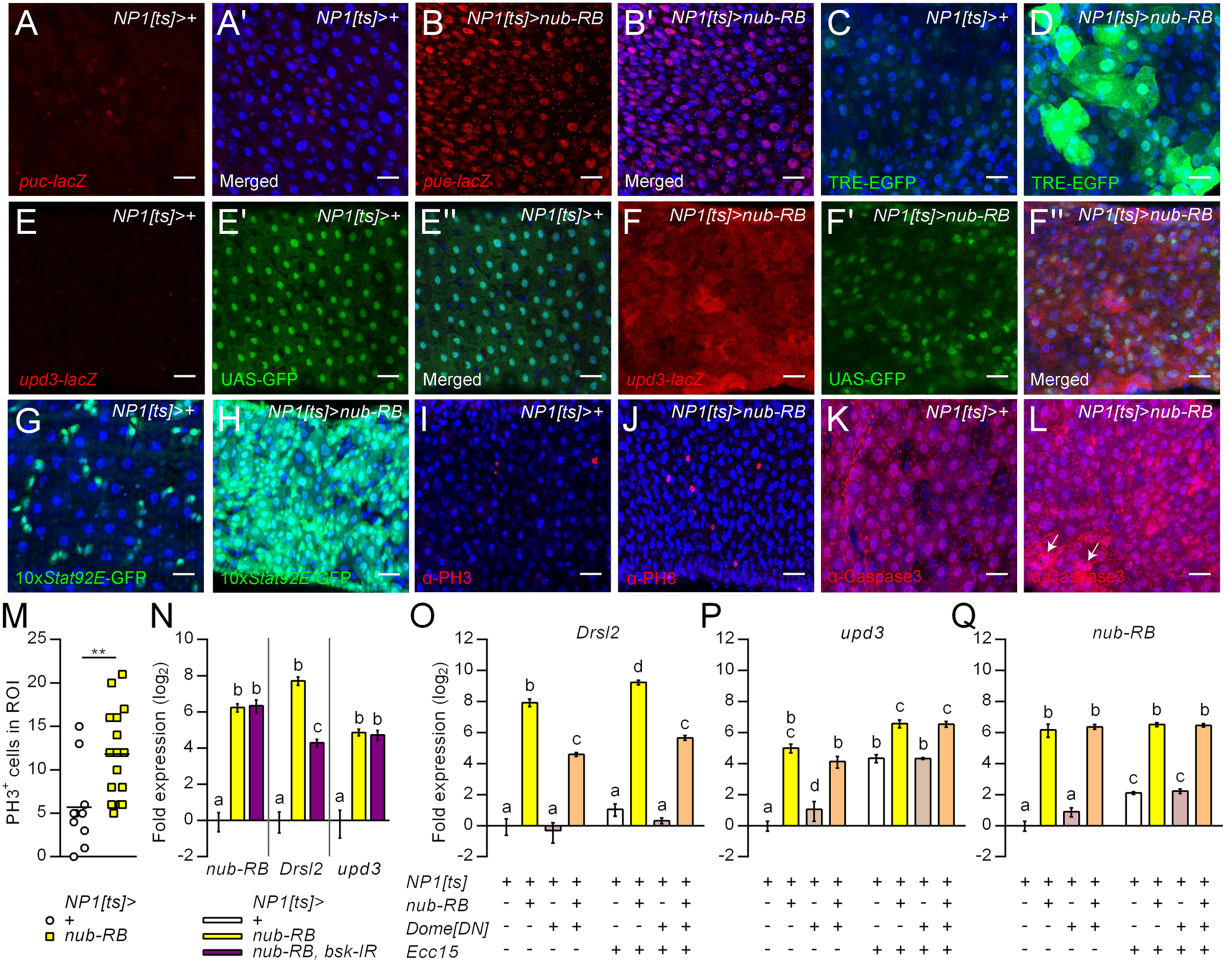
Prolonged *nub-RB* overexpression promotes a proinflammatory signature in the midgut. (A-L) Immunostainings of adult female posterior midguts after five days of *nub-RB* overexpression using *NP1-Gal4*^*ts*^ compared to driver controls. β-gal staining to highlight *puc*-*lacZ* expression (red), a target of the JNK pathway in controls (A-A’), or overexpression flies (B-B’). TRE-GFP (C-D’; green) was additionally applied to confirm JNK-signaling. Enterocyte morphology (green) and *upd3*-*lacZ* specific β-gal staining (red) were determined using flies co-expressing UAS-GFP and *upd3*-*lacZ* (E-F”). STAT activity was assayed using the reporter 10x*Stat92E*-GFP (G-H; green). Mitotic cells were stained with an antibody directed against PH3 (I-J; red). Cellular apoptosis was detected with α-caspase3 (K-L; red). Channels were merged to depict DAPI-stained nuclei (A’, B’, C-D, E”, F”, G-L; blue). (M) Quantification of PH3 positive cells in the R5 region of the midgut [57]. (N) Expression of selected transcripts from midgut extracts following Nub-PB overexpression alone (yellow) or combined with RNAi against *bsk* to block JNK signaling (purple), assayed relative to control levels (white; set to 1) by RT-qPCR. (O-Q) Expression of selected transcripts from midgut extracts following overexpression of *nub-RB* (yellow), UAS-dome^DN^ (gray; to inhibit JAK/STAT signaling) or both (orange), assayed relative to control levels (white; set to 1) by RT-qPCR. The experiment was performed on uninfected flies (-) or 24 h post oral infection with *Ecc15* (+). Asterisks and distinct letters (*p*<0.05) denote significant differences (*p<0.*05*; **p<0.*01*; ***p<0.*001*). Bars and boxes denote means ± SE (*N* = 3).

**Fig 7:**
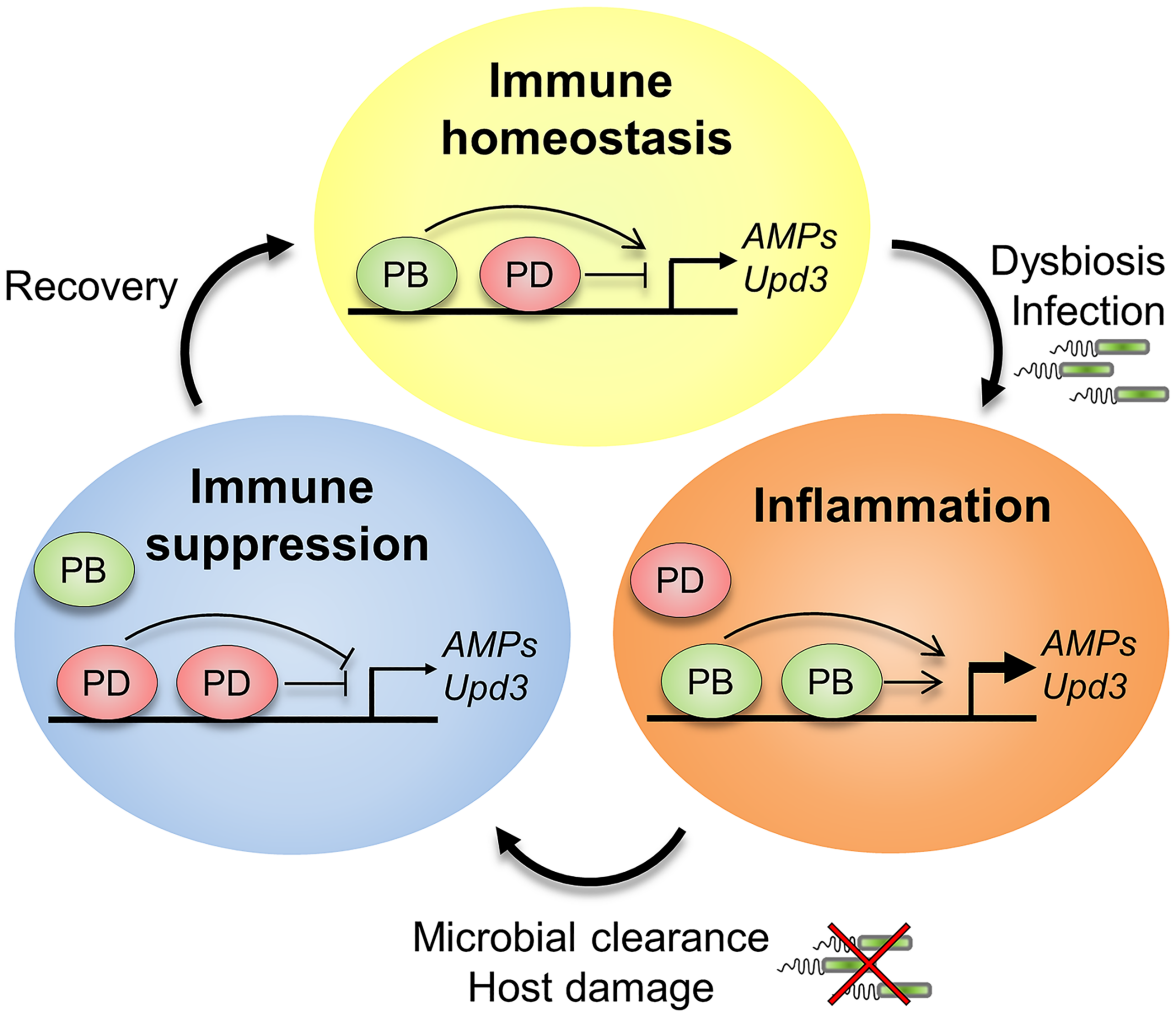
Model of the antagonistic actions of Nub isoforms. A balance between Nub isoforms is required to ensure immune homeostasis in the gut. During normal conditions, Nub-PD interacts with the proximal promoter region of immune-regulated genes to repress aberrant expression. Microbial dysbiosis or oral infection skews the isoform ratio towards Nub-PB, which through an unknown mechanism outcompetes Nub-PD and activates immune gene transcription. Once microbial homeostasis has been reestablished, the equilibrium between the isoforms is regained to balance gut immunity. Uncontrolled expression of Nub-PB or a lack of Nub-PD results in a hyperactivate immune response, loss of tissue homeostasis and early host death.

Among the identified IMD/Toll-independent DIRGs in the transcriptional profiles, several targets of the JAK/STAT pathway were induced, such as the gut-specific and infection-inducible *Drsl2*, stress-regulated *Turandots (Tots)* and the immunomodulatory cytokine *Diedel* (in the fat body), suggesting that Nub-PB either acts above, or at the level of, the JAK/STAT pathway (Fig 1F, S1 and S5 Tables) [10, 29]. In *Drosophila* gut enterocytes, the JNK pathway has been implicated in the regulation of Upd3, which in turn acts as a ligand for the JAK/STAT pathway in response to bacterial infection and stress [31]. Pathway activation triggers intestinal stem cell differentiation and proliferation to replenish extruded enterocytes [31]. Our observations that the expression of *upd3* and *Drsl2/3* is regulated by Nub-PB led us to investigate the role of the above-mentioned pathways in this context (Fig 6). Prolonged *nub-RB* overexpression for five days resulted in prominent induction of reporters for JNK (Fig 6A–6D), *upd3* (Fig 6E–6F”) and JAK/STAT (Fig 6G and 6H). This was accompanied by a general disorganization of enterocytes (Fig 6E’ and 6F’), increased number of mitotic cells (Fig 6I, 6J and 6M) and apoptosis (Fig 6K and 6L). Combined overexpression of *nub-RB* and targeted RNAi against the JNK-homolog *basket* (*bsk-IR*) in gut enterocytes attenuated the induction of *Drsl2* but not *upd3*, suggesting a dependency on JNK pathway activity for the former, but not the latter target (Fig 6N). To investigate the role of JAK/STAT, *nub-RB* was co-expressed together with a dominant negative form of the receptor Domeless (Dome^DN^; Fig 7O–7Q). Similarly to the findings in Fig 6N, and independent on infection status, this diminished the induced expression of *Drsl2*, but not *upd3*. Nub-PB might hence act together with the transcription factors of the JNK and JAK/STAT pathways to induce midgut-specific immune genes not typically regulated by NF-κBs. In support of this, *Drsl2* was found to contain putative DNA-binding motifs for Nub [15], AP-1 and Stat92E [33] in the proximal promoter region (Fig 2F). As expected, the expression of *nub-RB* was similar between the overexpression genotypes and was, in line with observations in Fig 6H, also induced by *Ecc15* infection in the driver control line (Fig 6N and 6Q). Together these results indicate that Nub-PB is sufficient to drive most, if not all, of the documented aspects of intestinal immunity and inflammation.

## Discussion

We have shown that the large isoform encoded by *nub*, Nub-PB, is a novel and exceptionally strong transcriptional activator of immune genes in *Drosophila*. Compared to the major immune regulatory factors, Rel and Dif, Nub-PB can potentially target an even broader set of DIRGs (Fig 1F). This could be explained by the notoriously promiscuous nature of Oct factors in terms of their conformations, dimer formations, protein-protein interactions and post translational modifications [21]. In humans, the Nub homolog, Oct-1, has been proposed to act as a switchable stabilizer of either repressed, induced or poised states of genes depending on its protein-protein interactions [34]. Furthermore, interactions between human Oct-1 and NF-κB have been demonstrated biochemically [35]. In agreement, we found that Nub-PB and Rel synergistically induce *CecA1* transcription (Fig 2C and 2E). Moreover, the Nub binding sites (Oct sites), located in immediate proximity to the κB-sites in the proximal promoter region of *CecA1*, were required to both repress [15] and fully induce expression (Figs 3 and S3), suggesting that the isoforms bind the same motifs to antagonistically regulate the transcription of Rel-target genes (Fig 3I).

Several Oct family members encode alternative isoforms [36]. Knowledge of their unique functions is, however, sparse. *Drosophila* appears to be no exception as both *nub* and its paralogs *pdm2/miti* and *pdm3* have similar gene organizations and encode promoter-specific isoforms with unknown functions. The underlying mechanism of the antagonism demonstrated in this study remains to be deciphered and is likely under multilayered regulation through isoform expression, mRNA/protein stability and the potential to form homo- and heterodimers. The very short unique N-terminus of Nub-PD implies that the domains required for transcriptional activation, e.g. *via* protein-protein interaction, are located in the larger N-terminal part of Nub-PB, although prediction analyses of protein functions did not reveal any distinct domains. Nub-PD might act as a passive repressor in a competitive manner, by binding to target sequences and prevent recruitment of additional factors, or alternatively form inactive heterodimers with Nub-PB. In accordance, isoform dimerization of the human POU protein Brn results in its inactivation [37]. Opposing effects of rat Oct-2 isoforms *in vitro* have also been reported and were dependent on the sequence and position of the octamer motif [38]. Finally, the human Oct-1 locus encodes at least three N-terminally distinct isoforms, which have been reported to act in partly distinct, albeit not opposite, manners [39]. It is hence plausible that distinctly acting, or even antagonistic isoforms of POU/Oct factors are an evolutionarily conserved phenomenon.

Intestinal immune and stress responses need to be tightly controlled to avoid excessive damage to host tissues. The gut microbial level and composition in adult flies correlate with host immunity and lifespan [6, 40, 41]. Age-related gut dysfunctions have been linked to dysbiosis, chronic inflammation and ultimately host death [42]. In line with these studies, we have previously observed that flies with the *nub*^*1*^ mutation (disrupts Nub-PD, but not Nub-PB expression) display chronic immune activation, microbial dysplasia and shortened lifespan [15, 23]. Importantly, we found that both isoforms are expressed in midgut enterocytes (Figs 4A–4D and S5B) and that overexpression of Nub-PB in these cells resulted in overall similar phenotypes as those previously found in *nub*^*1*^ mutants (Fig 4E, 4F, 4H and 4I), suggesting that the function of Nub-PD in this context is to counteract Nub-PB and repress aberrant responses. In fact, prolonged enterocyte-specific overexpression of Nub-PB was sufficient to drive most aspects of immune and inflammatory responses previously recorded during oral infection [31] including JNK and JAK/STAT pathway activation, Upd3 induction and increased gut mitosis and apoptosis (Fig 6). It is hence plausible that Nub-PB represents a missing node in the Upd3 mediated signaling from enterocytes to intestinal stem cells resulting in subsequent activation of JAK/STAT-driven stem cell proliferation and epithelial renewal [31], although a recent study demonstrated that a large number of transcription factors could potentially be involved [43]. The shortened lifespan of these flies correlated with a decreased microbiota (Fig 4F, 4H and 4I) and enhanced clearance of orally administered *Ecc15*, indicating that death is not a direct consequence of bacterial overgrowth but rather occurs due to a hyperactive immune response. In agreement, genetic manipulations to inhibit feedback regulation of the IMD pathway impair host lifespan and survival to infection [44–45]. Also, similar to *nub*^*1*^ mutants [23], microbial depletion extended longevity in *nub-RB* overexpressing flies, which furthermore suggests that bacterial exposure could aggravate the inflammatory response triggered by imbalance between Nub isoforms (S6B Fig). Enterocyte-specific and adult-restricted RNAi of *nub-RB* yielded overall the opposite phenotypes: reduced DIRG transcription, increased level of gut bacteria and enhanced lifespan, suggesting that Nub-PB is both necessary and sufficient to drive these phenotypes (Fig 4C and 4G–4I).

Importantly, genes involved in mounting an immune response at all levels from recognition to effectors and cytokines were activated by Nub-PB (Fig 1F). In contrast to Rel-driven immune responses [2], very few components providing negative feedback regulation were induced by Nub-PB. We hence speculate that endogenous Nub-specific transcription might be regulated *via* feedback on the Nub isoforms *per se*, possibly through protein degradation, a typical feature of many transcription factors. In addition, the combined effect of Nub-PB overexpression and infection resulted in immune gene expression levels up to three orders of magnitude above those of infected control flies, which implies that Nub-PB can enhance transcription during infection. Such vast expression levels are likely to impact host tolerance to microbial exposure [46] through loss of homeostasis, generation of self-inflicted damage, stress or even a metabolic collapse, which may explain the shortened life span and hypersensitivity to infection. Interestingly, Oct-1-deficient mouse fibroblasts are hypersensitive to stress [47], of which some parallels can be drawn to Nub-PB overexpressing, as well as *nub*^*1*^ flies [15, 23]. We propose that Nub-PD, in analogy with to Oct-1, acts as a stress sensor to neutralize the activity of Nub-PB, and that a balanced ratio between the isoforms is required to maintain a healthy gut environment.

Our finding that two N-terminal isoforms of the Oct-1/Oct-2 homolog, Nub, play antagonistic roles in immune/stress gene transcription provides genetic evidence of a novel switch-like regulation mediated *via* the same gene. We further suggest that Nub-PB and Nub-PD together form a molecular rheostat that dynamically tunes the transcriptional output to balance responses and efficiently eradicate pathogens while avoiding excessive activation and autoimmune-like reactions. This raises the possibility that Nub protein isoforms also regulate other physiological and developmental processes in opposite directions. Furthermore, the findings highlight a potential need to scrutinize the present view of POU/homeodomain networks by considering the presence of antagonistically operating isoforms, which may radically alter the transcriptional output. It remains to be explored whether similar modes of molecular rheostasis constitute a general and evolutionarily conserved mechanism to ensure flexible adjustment to environmental and developmental cues.

## Materials and methods

### Fly genotypes

The following transgenic fly lines were used in this study. (A) *w*^*1118*^;; (RRID:BDSC_5905) was applied as wild type. (B) y^*1*^w*; *nub*^*MI05126*^ (RRID:BDSC_37920). In this stock, the MiMIC cassette [48] is inserted into the 5’ UTR of *nub-RB* and the GFP expression derived from the MiMIC cassette is under control of the endogenous *nub-RB* promoter. (C) w*; *nub*^*AC-62*^; *UAS-mCherry/TM3* (RRID:BDSC_38418). The *nub*^*AC-62*^ allele carries a Gal4 enhancer trap inserted into the upstream region of *nub-RD* promoter, and has been recombined with nuclear *UAS-mCherry* (Müller and Affolter, personal communication to Flybase). (D) w*; *nub*^*GAL4*.*K*^ (RRID:BDSC_42699). The *nub*^*Gal4*.*K*^ line carries a Gal4 reporter driven by a 5.3 kb fragment from the *nub-RD* promoter and approximately 5 kb of upstream sequence[49]. *CecA1-lacZ* reporter lines: (E) *pA10* [50] and (F) *pA10ΔOct*, (this work). Lines for overexpression of transcription factors: (G) *w*;; *UAS-nub-RD* (15), (H) *w*; *UAS-nub-RB* (this work), (I) *w*;; *UAS-Dome*^*DN*^*/TM3*, *sb*^*1*^ (a gift from Nicolas Buchon). Lines for RNAi (J) *w*; *UAS-nub-RB-IR*^*KK113120*^ (RRID:FlyBase_FBst0476872, no predicted off-targets), (K) *y*^*1*^, *v*^*1*^;; *nub-RD-IR*^P{TRiP.JF02973}attP2^*/TM3*, *sb*^*1*^ (RRID:BDSC_28338; moved to a *w*^*1118*^ background prior to experiments), (L) w*;; *UAS-bsk-IR*. Gal4 driver lines for expression in specific tissues: (M) *w*^*1118*^; *c564-Gal4* (RRID:BDSC_6982) and (N) *w*^*1118*^; *c564-Gal4; tub-gal80*^*ts*^ for fat body and midgut, and (O) *w*; *NP1-Gal4* (a gift from Bruno Lemaitre) combined with *Tub-gal80*^*ts*^ or (P) *w*; *NP1-Gal4*; *Tub-gal80*^*ts*^, *UAS-GFP* (a gift from Nicolas Buchon) for midgut enterocytes and (Q) *w*;; *nub-RD-Gal4*^*VT006452*^ (RRID:FlyBase_FBst0483181). Mutant fly strains: (R) *w*;; *Rel*^*E20*^, *e*^*s*^ (RRID:BDSC_9457). (S) *w*; *UAS-nub-RB*; *UAS-nub-RD* was constructed from (G) and (H); (T) *w*; *UAS-nub-RB*; *UAS-Dome*^*DN*^ from (H) and (I); (U) *w[1118]*; *UAS-nub-RB*; *UAS-bsk-IR* from (H) and (L); (V) *w*^*1118*^; *c564-Gal4*; *Rel*^*E20*^, *e*^*s*^ from (M) and (R); [23] *w*^*1118*^; *UAS-nub-RB*; *Rel*^*E20*^, *e*^*s*^ from (H) and (R) using the double balancer line (W) *w*^*1118*^; *if/CyO*; *MKRS/TM6B*, *tb*^*1*^. (X) *w*;; TRE-eGFP, (Y) *w*;; *puc-lacZ*/*TM3* (gifts from Ulrich Theopold /Dirk Bohmann), (Z) *w*; *upd3-lacZ* and (AA) *w*; 10x*Stat92E*-GFP (gifts from Nicolas Buchon) were recombined to (I) and crossed to (O) or (P) for immunostainings.

### Fly rearing

Flies were maintained on instant potato medium [23] in mixed female/male populations at 25 °C, 60% RH, with a 12 h light/12 h dark cycle. For experimental crosses, flies were reared at 18 °C until at least two days post eclosure, and then switched to 29 °C. Overexpression experiments were carried out following two days of incubation at 29 °C. For RNAi, flies were maintained at 29 °C for 5–7 days prior to experiments. All experiments were performed using 5–10 day old females with the exception of lifespan (newly eclosed males and females, which were recorded daily) and survival assays post infection (5–10 day old males and females maintained separately). For lifespan analysis in germ free conditions, food was supplemented with an antibiotic cocktail [23].

### Cell culturing

*Drosophila* mbn-2 cells (DGRC, cat. no. 147) were cultured in Schneider’s medium (Gibco) supplemented with 10% fetal bovine serum (Gibco) in 5 ml plates at 25 °C, to a cell density of approximately 6–7 × 10^6^ cells/plate.

### Creation of UAS-nub-RB transgenic flies

Total RNA was isolated from *OrR* males with TRIsure (Bioline) and used as template for reverse transcriptase (RT). Due to the gene locus size, two RT-PCR reactions were performed in parallel, using coupled AMV RT and Tfl DNA polymerase (Access RT-PCR system, Promega Biotech AB), to amplify the 5’ and 3’ fragments of the *nub-RB* cDNA separately. The 5’ forward primer was constructed with a NotI site-containing overhang and an internal reverse primer; the reverse with an internal forward primer and a reverse primer with a *BamHI* site-containing overhang at the 3’-end of the *nub-RB* cDNA. The two cDNAs, 1603 bp and 1803 bp respectively, which partly overlap and contain a common *EcoRV* site in exon 5, were cloned into the pGEM-T easy vector. After DNA sequence verification, the two *nub-RB* cDNA halves were excised with NotI/EcoRV and EcoRV/BamHI respectively and then ligated into the *pcDNA3*.*1(-)* vector to create the complete 2883 bp *nub-RB* coding sequence with short 5’ and 3’ UTRs. *Drosophila* expression plasmids were created using Gateway Technology (Invitrogen, Carlsbad, CA, USA). Briefly, *nub-RB* coding cDNA was amplified from *pcDNA3*.*1(-)nub-RB* using *Pfu* DNA polymerase (Thermo Fisher Scientific, Waltham, MA, USA) according to standard procedures. The purified PCR product was cloned into the pENTR/D-TOPO vector using pENTR Directional TOPO Cloning (Invitrogen) followed by recombination of the *nub-RB* cDNA into the *pTW* and *pAW* destination vectors (obtained from TD Murphy) using LR Recombination and the LR Clonase enzyme mix (Invitrogen). P element transformation of *w*^*1118*^ flies with *pTW-nub-RB was* performed according to standard procedures[51]. Transfection of cells with *pAW-nub-RB* is described below.

### CecA1-luciferase and CecA1-lacZ reporter constructs and flies

Plasmids and transgenic fly lines with *CecA1-luciferase* and *CecA1-lacZ (pA10)* reporter constructs with the complete *CecA1* upstream region and a short 5’ UTR have been described previously [13, 47]. To create a *CecA1-lacZ* reporter with the Oct cluster deleted, *pA10* was used as template for inverse PCR amplification with phosphorylated primers and cloned as described previously for *CecA1*Δ*Oct-luc* [15]. The whole *CecA1*Δ*Oct-lacZ* fragment was thereafter excised by XbaI-XhoI digestion and ligated into the P element vector *pW8* plasmid, opened with the corresponding enzymes. P element-transformation of *w*^*1118*^ was carried out according to standard procedures [51].

### Cell transfections

Transfections were performed with 1 μg of *pA10-luc* construct and mixed with 1 μg of *pAW-nub-RB*, 500 ng of *pAct-Rel*, and 100 ng of Pol III-Renilla luciferase (Addgene plasmid 37380) as internal reference. Carrier DNA was added to reach 10 μg in each sample, and transfections were performed using a calcium phosphate transfection kit (Invitrogen) as described previously [15]. Luciferase values were measured by the Dual-luciferase Reporter Assay System (Promega). To stimulate immune activation, peptidoglycan was added in the form of a crude lipopolysaccharide (LPS) preparation (25 μl of 2 mg/ml), 4 h prior to harvest.

### Reporter gene expression in transgenic flies

For analysis of *CecA1-lacZ* reporter gene expression in transgenic flies, adults were dissected to remove heads and separate the digestive system from the rest of the fly, then fixed in 1% glutaraldehyde in phosphate-buffered saline and stained for β-gal activity using 5-bromo-4-chloro-3-indolyl-β-D-galactopyranoside (X-gal) as substrate, as described previously [52]. Incubation with substrate was done for 2 h at 37 °C, and in some cases continued for 16 h at 25 °C. To estimate X-gal intensity, 8-bit images were first processed in Adobe Photoshop (version 2015 CC) using the grayscale tool to convert all colors to white except blue (converted to grayscale). Mean gray values per fly were subsequently measured in ImageJ (in the range 0 (white, no staining) to 255 (black)) using the freehand tool (*N* = 6).

### RNA extraction and quantitative RT-qPCR analysis

Total RNA extractions were carried out using TRIsure (Bioline) from adult females (three flies for whole fly extracts and six dissected tissues, respectively, per replicate), followed by DNAse treatment and cDNA synthesis as previously described with a few modifications[15]. Taqman probes/primers were used to measure gene expression according to the manufacturer´s instructions (Applied Biosystems). Primer/Probes: *nub-RD* (CG34395-PD): Dm01841366_m1 (Applied Biosystems); *nub-RB* (CG34395-PB): Dm01812808_s1 (Applied Biosystems). Primer/probes for AMP gene expression were as previously published [15]. Samples were analyzed in biological triplicates or quadruplicates and relative amounts of each target were quantified relative to a set standard curve pooled from all samples in the analysis and finally normalized relative to those of *RpL32*.

### Microarray analysis, processing and extended analysis

Overexpression of *nub-RB*, was carried out in flies of the genotype *w; UAS-nub-RB/c564-Gal4* and compared with flies carrying the *c564-Gal4* driver but no UAS target gene (*w; c564-Gal4/+)*. Flies were raised at 18 °C and adults reared in mixed sex populations at 25 °C. Female flies were used at 10 days of age, dissected to separate guts and the rest (flies without guts and heads). Total RNA extractions were carried out using TRIsure (Bioline), followed by DNAse treatment with Turbo-DNase (Ambion) and purification using RNeasy (Qiagen). Tissues (25 per replicate) from three or four independent pools of flies were analyzed as biological replicates on *Drosophila* Agilent microarrays. Total RNA (150 ng) was amplified and labeled using Low Input Quick Amp Labeling Kit according to the manufacturer’s instructions. Cyanine 3-CTP-labelled c-RNA (1.65 ug) was used for 17 hour of hybridization at 65 °C to the *Drosophila* (V2) Gene Expression Microarray, 4x44K. The hybridized arrays were washed and scanned with the Agilent DNA microarray scanner G2505C. The fluorescent intensities of the scanned images were extracted and preprocessed using the Agilent Feature Extraction Software (version 10.7.3.1). Preprocessing of the raw data was done according to the standard analysis pipeline at the Bioinformatics and Expression Analysis Core Facility at Karolinska Institutet, Huddinge, Sweden. In short, Agilent processed signals (i.e. feature gProcessedSignal) were imported to Partek Genomics Suite and subjected to quantile normalization. After preprocessing and normalization, the data was filtered to remove genes that were not expressed at detectable levels (estimated background signal). A factorial map of principal component analysis was executed on the whole expressed data using Bioconductor 3.1 and R 3.1. Multiple T-tests of the entire dataset were performed in Graphpad Prism 6 and *p*-value thresholds adjusted using the FDR approach (Q set to 1%). Filtered data (above detection limit in at least one of the groups compared and below the adjusted *p-value* threshold) were further used throughout the extended explorative downstream analysis with a few exception were a subsequent fold change cut-off were additionally applied (where denoted). The GSEA to reveal enriched GO biological processes was performed using Cytoscape (version 3.6.0) and the plugin Bingo (version 3.0.3). The analysis was executed using the hyper-geometric test with Benjamini-Hochberg FDR correction. Venn diagrams were constructed using the web-based software Venny (version 2.1).

### Immunostainings

Female guts were dissected in PBS, pH 7.4 and fixed with 4% paraformaldehyde (PFA) at room temperature for 1 h. Following two 5 min washes in PBS-T (1x PBS +0.1% TritonX-100), tissues were incubated in blocking solution (PBST+ 0.5% normal goat serum) at room temperature for 30 min and probed with primary antibodies (mouse α-β-gal [1:20; DSHB, RRID:AB_528101]; rabbit α-PH3 [1:300; Millipore, RRID:AB_310177]; rabbit α-cleaved caspase-3 [1:300; Cell Signaling, RRID:AB_2341188]) at 4 °C over night. The next day, tissues were washed in PBST 4**×**15 min, incubated with secondary antibodies (goat α-mouse Alexa594 [1:1000; Invitrogen, RRID:AB_141372]; goat α-rabbit Alexa594 [1:1000; Invitrogen, RRID:AB_141359]) at room temperature for 2 hours, washed again in PBST 4**×**15 min and then stained with DAPI (Sigma) at room temperature for 10 min. Stained samples were mounted on a glass slide with DABCO (Sigma) and confocal images were acquired using a LSM 780 microscope (Zeiss).

### Western blot

Carried out as previously described with a few modifications [15]. Briefly, whole fly extracts were prepared by grinding three flies per replicate in 2x standard Laemmli buffer in a 1.5 ml microcentrifuge tube using a plastic pestle followed by 10 min heating at 70 °C and 15 min centrifugation at 4 °C to remove debris. Samples were run on 10% polyacrylamide gels (125V, 90 min), wet transferred to PVDF membranes (Millipore; 10V O/N followed by 40V for 1 h) and blocked with 5% non-fat dry milk in Tris-buffered saline, 0.1 Tween-20. Membranes were incubated with rabbit-α-Nub [15] in TBS-T at 4 °C overnight, washed and incubated for 1 h at RT with HRP-conjugated donkey-α-rabbit (1:10,000; GE Healthcare, cat. no. LNA934V/AG). Bands were obtained using SuperSignal West Pico (Thermo Fisher Scientific). After quick stripping (5 min protocol using 0.3 M NaOH), PVDF membranes were blocked and reprobed with mouse α-β-Actin (1:10,000; Abcam) followed by HRP-conjugated sheep-α-mouse (1:10,000; GE Healthcare, cat. no. LNA931V/AG) as loading control.

### Bacterial stocks and infections

*Erwinia carotovora carotovora 15* (*Ecc15*) and *Ecc15*-GFP, kindly donated by Bruno Lemaitre, were cultured in LB medium at 30 °C with shaking [53]. For oral infections, overnight cultures of bacteria were pelleted and resuspended to OD100 in a 1:1 ratio of bacterial medium and Milli-Q H_2_O with 5% sucrose and 1% isotonic phosphate-buffered saline (PBS), pH 7.4. Infection vials were prepared by depositing 100 μl of bacteria or control solution on a filter paper placed on top of a 1% PBS, 2% agar gel to maintain humidity. Prior to challenge, flies were starved and desiccated in empty vials for 2 h, then briefly anesthetized to allow transfer to infection vials. Flies were maintained on this diet for 1 h (bacterial count) or 24 h (survival analysis) and subsequently transferred to fresh food vials. For systemic infections, *E*. *cloacae* β12 was grown over night at 37 °C with shaking, pelleted and resuspended in PBS at OD 1. Flies were infected by abdominal injections with approximately 50 nl of bacterial suspension under brief anesthesia. Following all infections, flies were maintained at 29 °C. Smurf assay [54] was initiated at 16 hpi by transferring flies onto regular fly food supplemented with Coomassie Brilliant Blue FCF (commercial, food grade). Flies were maintained up to two weeks post infection on this diet and observed for smurfs daily.

### Bacterial quantifications

For relative 16S rDNA quantification of gut bacteria, midguts from six flies per replicate were dissected and bacterial DNA extracted using the DNA Blood and Tissue (Invitrogen) kit according to instructions, including the Gram^+^ lysis step. For bacterial counts of *Ecc-15* post infection, *Ecc15*-*GFP* was applied and cultured in LB with carbenicillin (50 μg/ml). Oral infections were performed as described above. At indicated time points, individual flies were anaesthetized and ground in 100 μl PBS on ice. Ten-fold serial dilutions were added to LA-carbenicillin plates and subsequently incubated over night at 30 °C. GFP-positive bacterial colonies were quantified from seven individual flies per replicate. The experiment was performed three times for conditional rearing, and once under germ free conditions.

### Capillary feeding

Carried out as described by Ja et. al with a few modifications [55]. Two females per replicate were placed in a vertically standing microcentrifuge tube with the bottom part excised and sealed with cotton to allow air exchange. Microcapillaries (3.2 mm, 5 μl; Drummond) were filled with a 5% sucrose, 5% yeast extract solution placed through a small hole in the cap of the tube. To prevent evaporation, tubes were maintained in a high humidity climate chamber, with quick replacement of microcapillaries every 24 h. Following 48 h entrainment at 18 °C, tubes were placed at 29 °C to induce Gal4-activity. For overexpression, measurements were performed between day 2–3; for RNAi between day 5–6 after the temperature shift.

### Quantification and statistical analysis

Analyses of two sample means were performed using a two-tailed Student’s unpaired *t*-test (Figs 4C, 4D, 5I–5L, 6M and S5B–S5D, S7B and S7C). Equal variances between the groups were ensured using an F-test (*p*>0.05). Multiple comparisons were carried out using a one-way (Figs 2A–2C, 3H, 4E–4G, 5N and 6N), or two-way ANOVA (Figs 2D–2F, 5C–5G and 6O–6Q) combined with Tukey’s post hoc test. In Fig 5C–5F, denoted significant differences between cohorts were derived from the interaction between the two factors: 1) time post infection and 2) *nub-RB* overexpression as determined by a two-way ANOVA without further post-hoc analysis. In Fig 5N, Dunnett’s test was applied to analyze significant fold changes post infection relative to at 0 hpi (set as control). Lifespan and survival assays were analyzed using Mantel-Cox log-rank test with Bonferroni-corrected thresholds for significance (*p*<0.05/number of comparisons). Normalized qRT-PCR data were log_2_-transformed in order to model proportional changes prior to statistical analysis. Statistical analyses and graph constructions were carried out in Graphpad Prism 6. For experiments involving dissections, sample sizes were set to *N* = 3–4 to allow collections within the designated time point and to minimize sample degradation due to handling. Parametric tests were chosen based on previous experience of the normality of gene expression of the selected targets. Fold changes in expression were quantified relative to the mean value of the control cohorts.

## Supporting information

S1 Fig Hierarchical clustering and Venn diagram of global transcription in Nub-PB overexpressing flies. (A) Hierarchical clustering was conducted using all transcripts expressed over background signal in at least one sample group after removing background and filtering for fold change ≥2. (B) Venn diagram depicting the overlap of differentially expressed genes by c564>*nub-RB* in “rest” and “gut” cohorts.
(TIF)

S2 Fig Gene set enrichment analysis (GSEA) of downregulated transcripts in respective tissue. The colored nodes corresponding to different Gene Ontology clusters were found with increasing statistical significance after Benjamini and Hochberg FDR correction (*p*<10^−3^) whereas non-colored nodes were not significant. Analyses were based on 382 and 386 probes from “Rest” and “Gut”, respectively.
(TIF)

S3 Fig Complementary figure to Fig 3. (A-F) β-gal staining in fat body and other tissues in male flies as readout of expression from the *CecA1*-promoter with (*pA10*) or without (*pA10ΔOct*) the Oct-cluster. See Fig 3 for details.
(TIF)

S4 Fig Visualization of Nub isoform expression in selected tissues by fluorescent reporters. (A-C”) Dual fluorescence assay of *nub-RB*-GFP (green) and *nub-RD*^*AC-62*^ (red) expression in larval proventriculus (A-A”), wing discs (B-B”), brain and leg discs (C-C”). Channels were also merged with DAPI overlay to depict nuclei (A”, B”, C”). (D-I) Adult expression of either Nub isoform reporter in adult abdomen (D-E), wing veins (F-G) and leg joints (H-I).
(TIF)

S5 Fig Nub-PB regulates immune gene expression in midgut enterocytes but not fat body. (A) Western blot of Nub from dissected midguts or corresponding carcasses in OregonR flies. (B) Western blot from whole fly extracts following five days incubation at 29 °C to achieve full effect of the NP1^ts^-Gal4-driven RNA interference of *nub-RB* (*nub-RB-IR*). Upper panel, representative immunoblot; lower panel, quantification of Nub-PB protein levels, normalized to β-Actin bands (loading control). Asterisks denote significant differences, determined by Student’s unpaired t-test (*p<0.05, *N* = 3). (C-D) qRT-PCR of *nub-RB* and *Drsl3* following c564[ts]-Gal4-driven RNAi of *nub-RB* in midguts (C) or abdominothoracic carcasses (D). Asterisks denote significant differences, determined by Student’s unpaired t-test (***p<0.001, *N* = 3).
(TIF)

S6 Fig Additional lifespan experiments including germ free conditions and statistics. (A-B) Lifespan analysis of females and males of the denoted genotypes. Flies were conditionally reared (CR) or maintained on antibiotic-supplemented food to become germ free (GF). The number of dead flies were recorded daily. (C) Statistics from the three individual experiments under CR conditions. Group letters denote significant differences (*p*_Bonferroni-corrected_<0.0083). (D) Relative lifespan extension in GF conditions for respective genotype (*p*_Bonferroni-corrected_<0.0125 was considered significant).
(TIF)

S7 Fig Additional data relating to Fig 5. (A-B) *Ecc15* counts and relative bacterial clearance in flies overexpressing *nub-RD* in enterocytes, compared to controls. (C) Feeding rates following *nub-RD* overexpression. See Fig 5 for details.
(TIF)

S1 Table Whole transcriptome comparison of the *c564>nub-RB* and *c564>+* cohorts. (XLSX)

S2 Table List of the 132 probes indicating differentially expressed transcripts in common for “rest” and “gut” cohorts. (XLSX)

S3 Table A list of all significantly enriched GO clusters and the associated differentially expressed transcripts in “gut”. (XLSX)

S4 Table Differentially expressed transcripts in “rest” belonging to the GO cluster “proteolysis”, filtered further by fold change ≥2 or ≤-2 (113 genes). (XLSX)

S5 Table Merged list of all 152 differentially expressed DIRGs annotated according to biological function (relating to Fig 1F). (XLSX)

S6 Table Comparison of immune gene expressions between *c564>nub-RB* and *nub*^*1*^ flies [15] in “rest”, both presented in fold changes relative to respective control. *, verified by RT-qPCR; italicized values, below threshold or not significant.
(XLSX)
