# Supplementary figures and images for: Nubbin isoform antagonism governs Drosophila intestinal immune homeostasis
Source: PLoS Pathog. 2018 Mar 2;14(3):e1006936. doi: 10.1371/journal.ppat.1006936 (PMC5851638; doi:10.1371/journal.ppat.1006936)

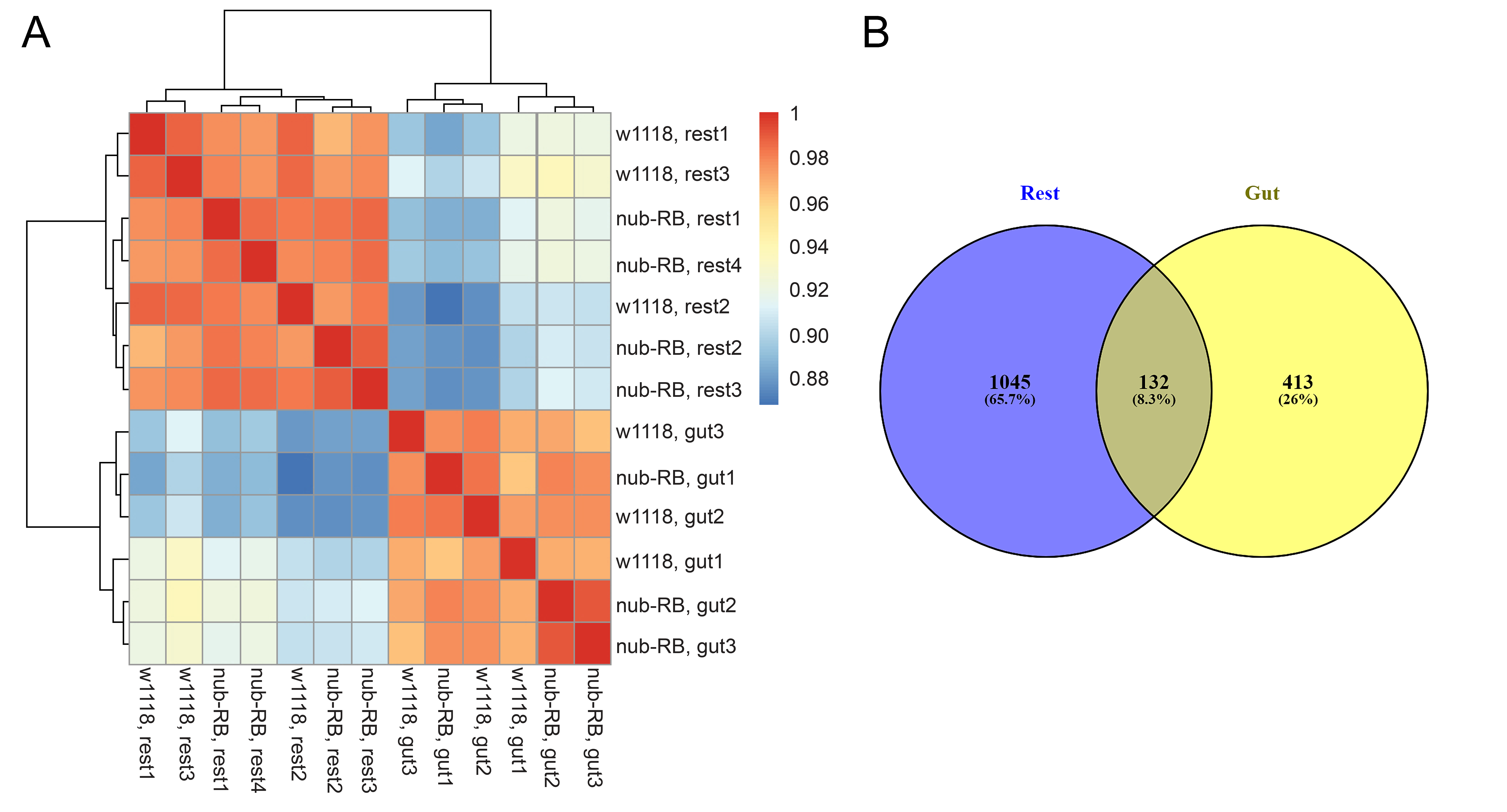

Supplement: S1 Fig — (A) Hierarchical clustering was conducted using all transcripts expressed over background signal in at least one sample group after removing background and filtering for fold change ≥2. (B) Venn diagram depicting the overlap of differentially expressed genes by c564>nub-RB in “rest” and “gut” cohorts. (TIF) [file ppat.1006936.s001.tif]

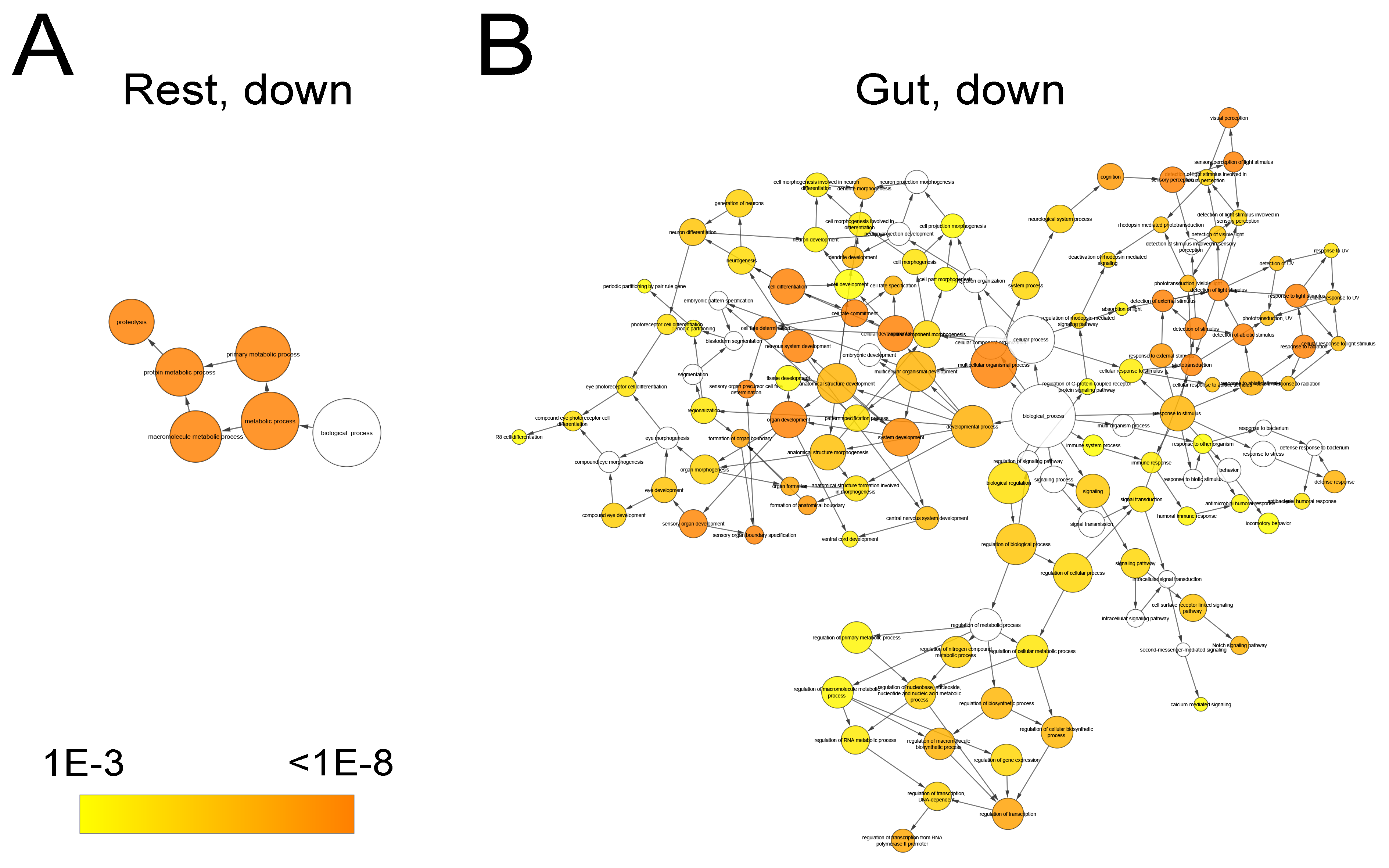

Supplement: S2 Fig — The colored nodes corresponding to different Gene Ontology clusters were found with increasing statistical significance after Benjamini and Hochberg FDR correction (p<10−3) whereas non-colored nodes were not significant. Analyses were based on 382 and 386 probes from “Rest” and “Gut”, respectively. (TIF) [file ppat.1006936.s002.tif]

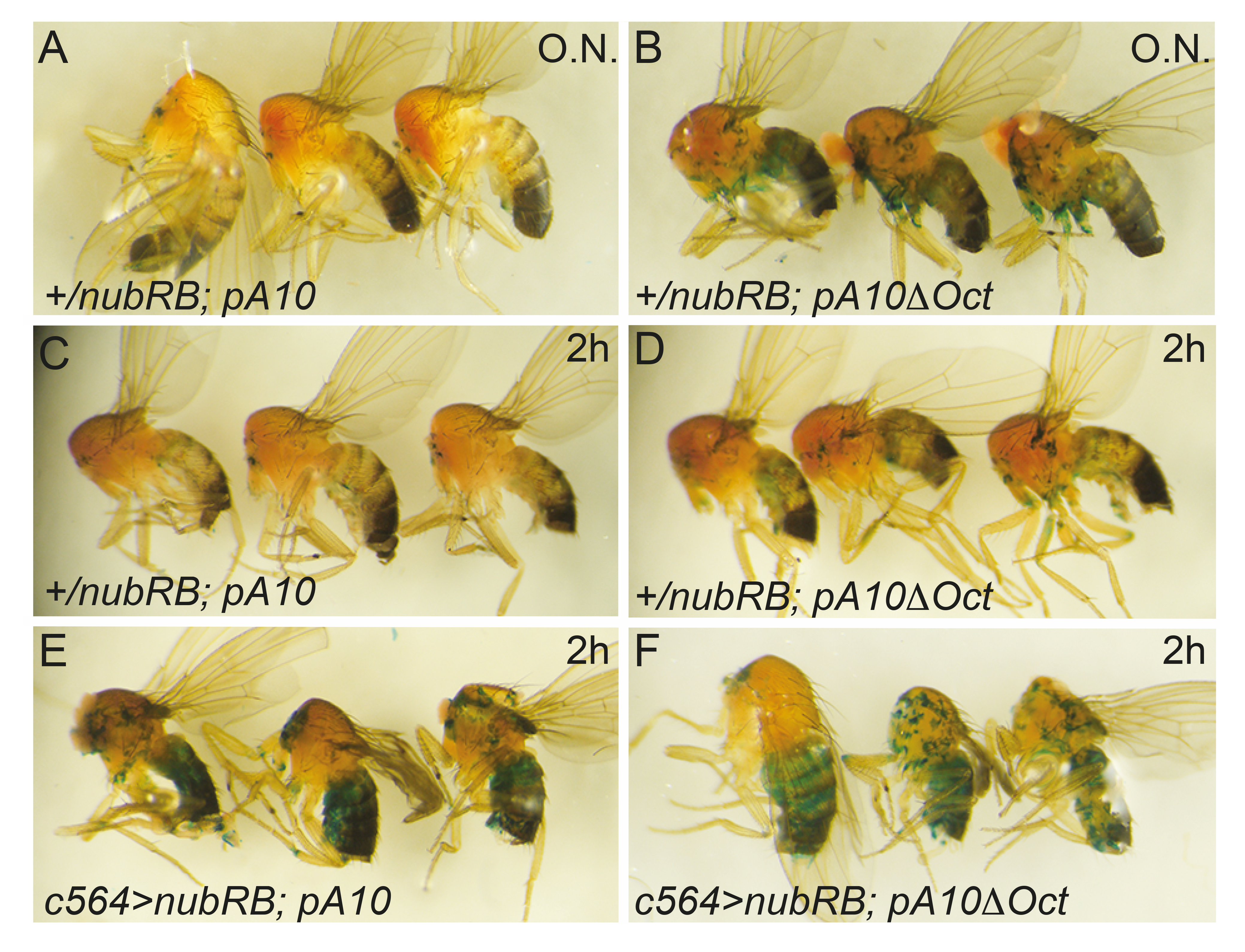

Supplement: S3 Fig — (A-F) β-gal staining in fat body and other tissues in male flies as readout of expression from the CecA1-promoter with (pA10) or without (pA10ΔOct) the Oct-cluster. See Fig 3 for details. (TIF) [file ppat.1006936.s003.tif]

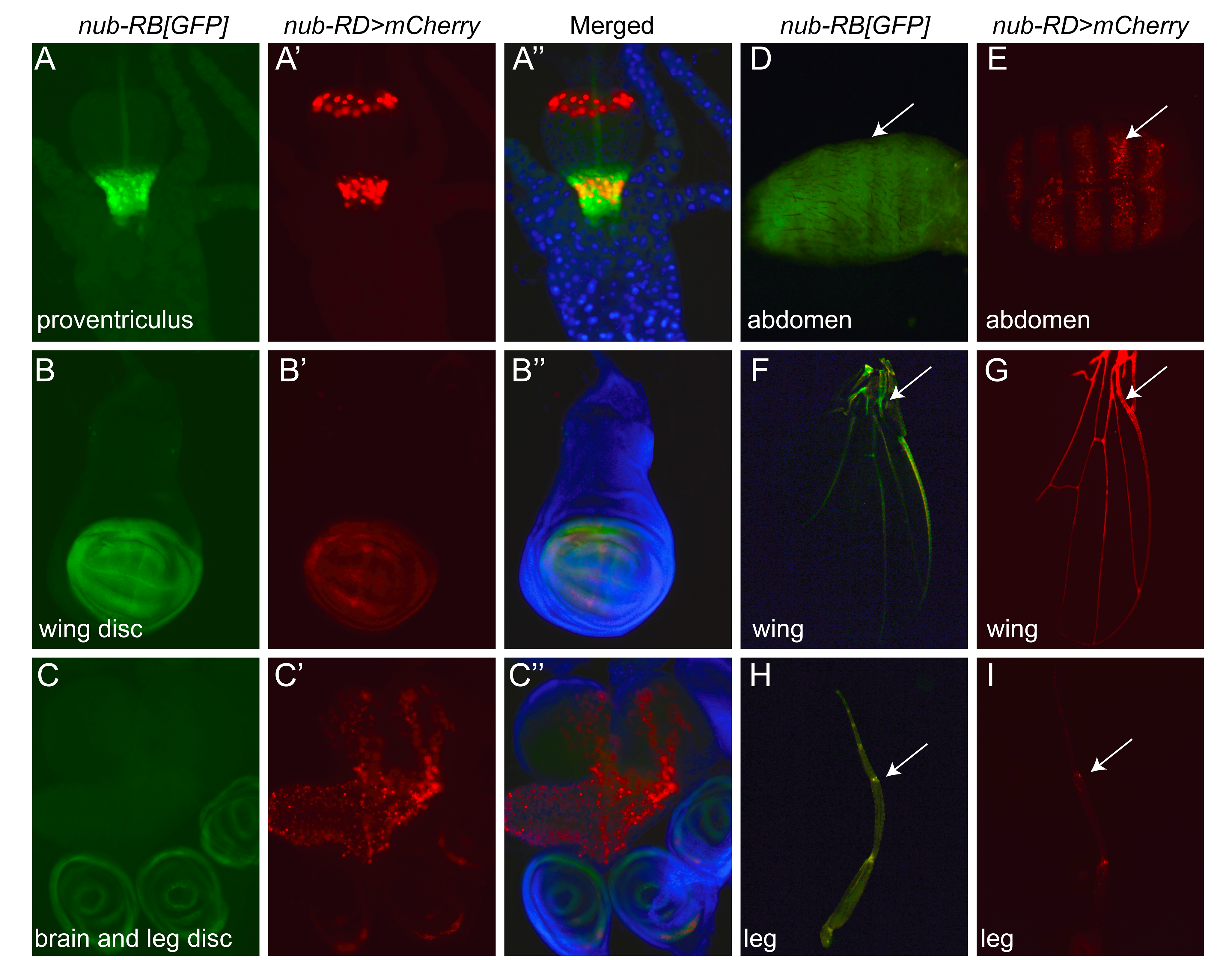

Supplement: S4 Fig — (A-C”) Dual fluorescence assay of nub-RB-GFP (green) and nub-RDAC-62 (red) expression in larval proventriculus (A-A”), wing discs (B-B”), brain and leg discs (C-C”). Channels were also merged with DAPI overlay to depict nuclei (A”, B”, C”). (D-I) Adult expression of either Nub isoform reporter in adult abdomen (D-E), wing veins (F-G) and leg joints (H-I). (TIF) [file ppat.1006936.s004.tif]

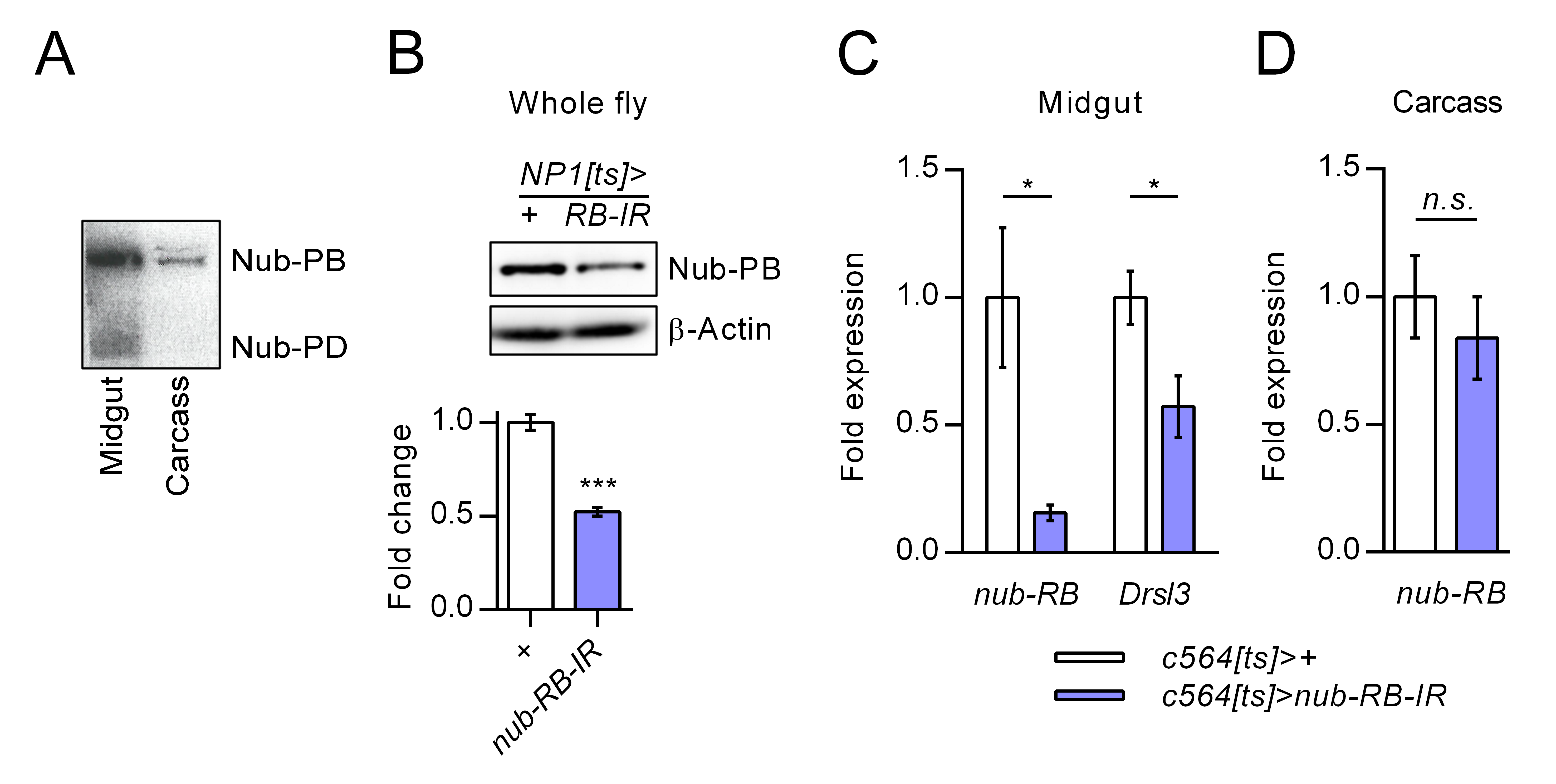

Supplement: S5 Fig — (A) Western blot of Nub from dissected midguts or corresponding carcasses in OregonR flies. (B) Western blot from whole fly extracts following five days incubation at 29 °C to achieve full effect of the NP1ts-Gal4-driven RNA interference of nub-RB (nub-RB-IR). Upper panel, representative immunoblot; lower panel, quantification of Nub-PB protein levels, normalized to β-Actin bands (loading control). Asterisks denote significant differences, determined by Student’s unpaired t-test (*p<0.05, N = 3). (C-D) qRT-PCR of nub-RB and Drsl3 following c564[ts]-Gal4-driven RNAi of nub-RB in midguts (C) or abdominothoracic carcasses (D). Asterisks denote significant differences, determined by Student’s unpaired t-test (***p<0.001, N = 3). (TIF) [file ppat.1006936.s005.tif]

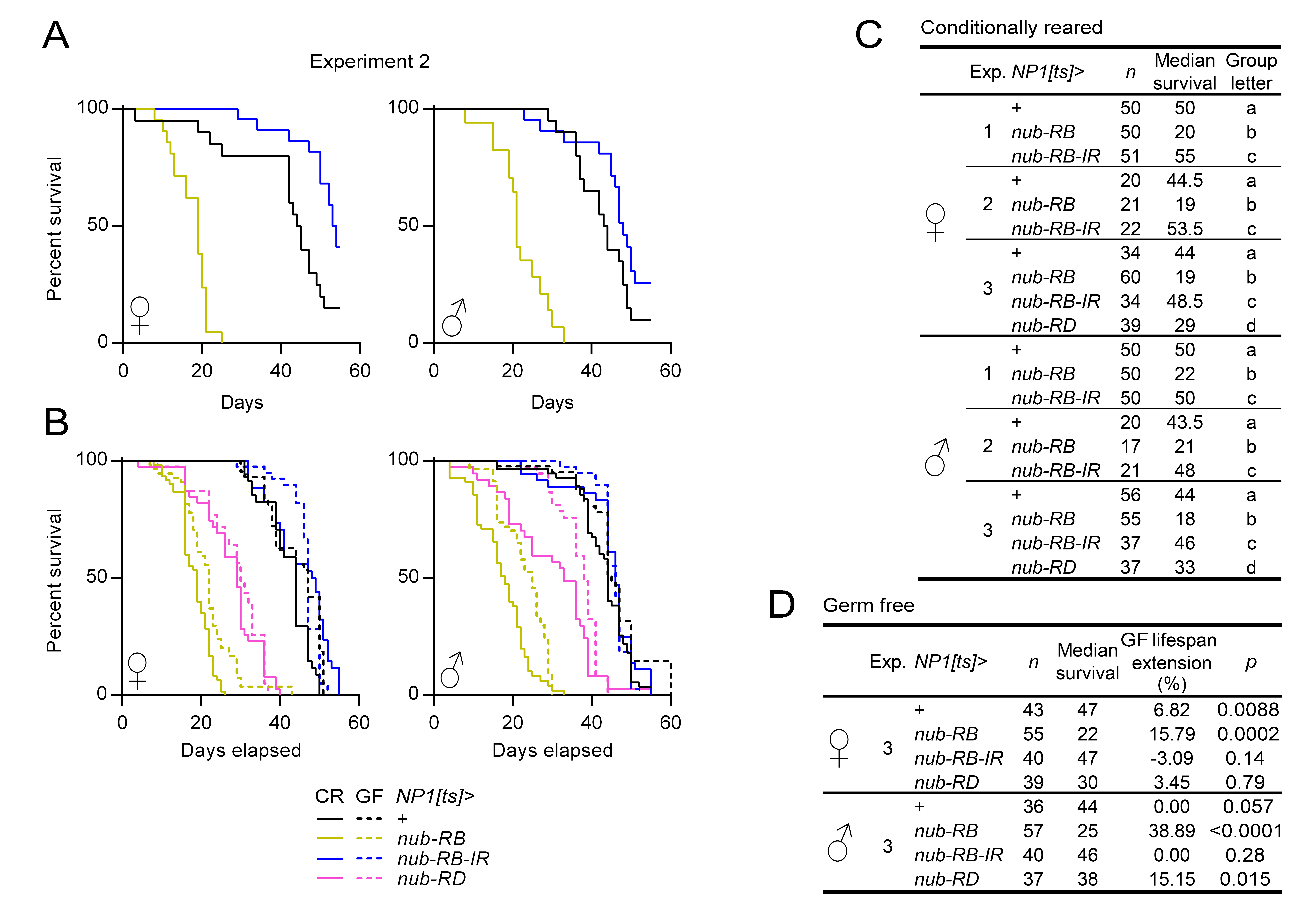

Supplement: S6 Fig — (A-B) Lifespan analysis of females and males of the denoted genotypes. Flies were conditionally reared (CR) or maintained on antibiotic-supplemented food to become germ free (GF). The number of dead flies were recorded daily. (C) Statistics from the three individual experiments under CR conditions. Group letters denote significant differences (pBonferroni-corrected<0.0083). (D) Relative lifespan extension in GF conditions for respective genotype (pBonferroni-corrected<0.0125 was considered significant). (TIF) [file ppat.1006936.s006.tif]

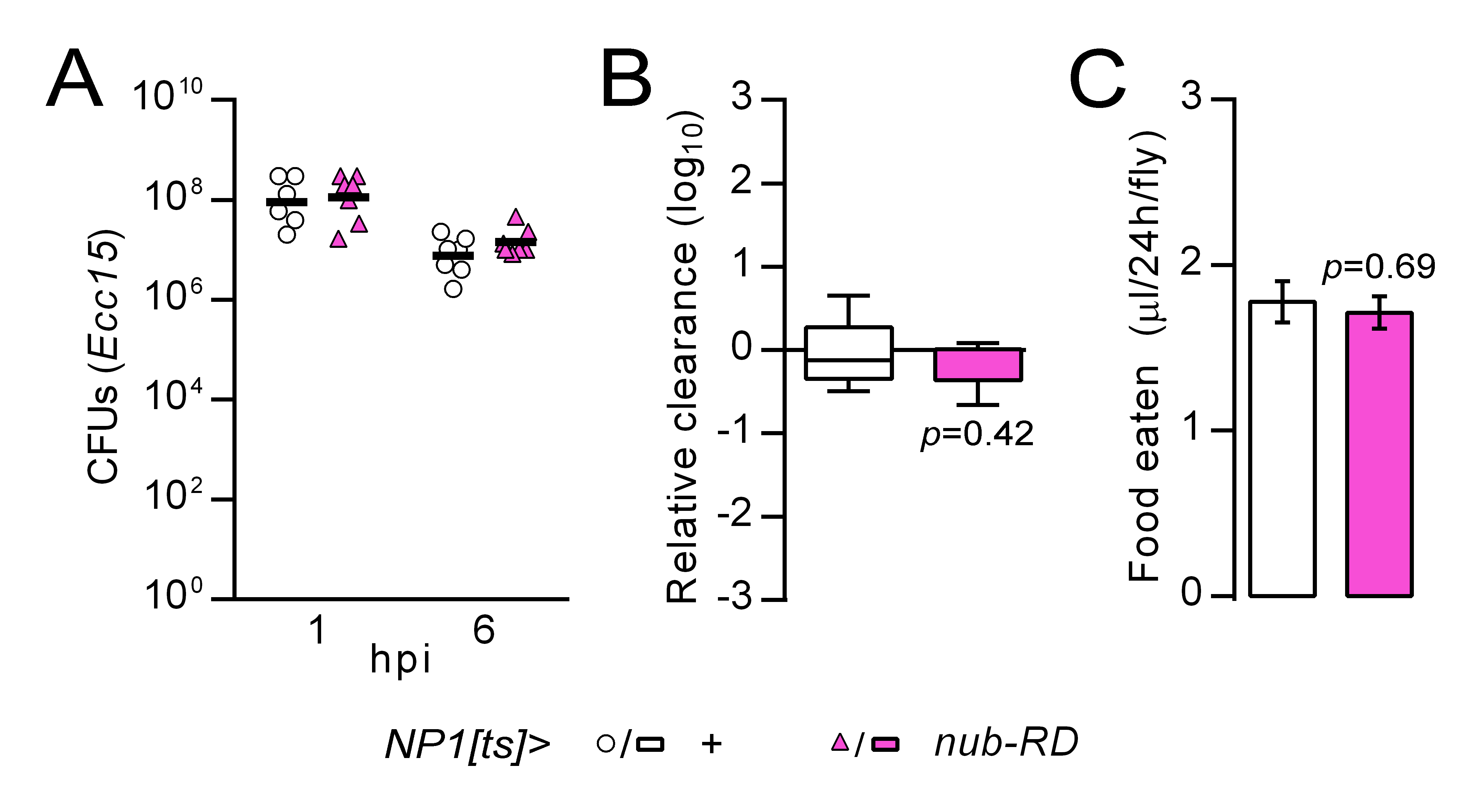

Supplement: S7 Fig — (A-B) Ecc15 counts and relative bacterial clearance in flies overexpressing nub-RD in enterocytes, compared to controls. (C) Feeding rates following nub-RD overexpression. See Fig 5 for details. (TIF) [file ppat.1006936.s007.tif]
